# A *BrLINE1-RUP* insertion in *BrCER2* alters cuticular wax biosynthesis in Chinese cabbage (*Brassica rapa* L. ssp. *pekinensis*)

**DOI:** 10.3389/fpls.2023.1212528

**Published:** 2023-07-12

**Authors:** Biyuan Li, Zhichen Yue, Xiaoya Ding, Yanting Zhao, Juanli Lei, Yunxiang Zang, Qizan Hu, Peng Tao

**Affiliations:** ^1^ Institute of Vegetables, Zhejiang Academy of Agricultural Sciences, Hangzhou, China; ^2^ Collaborative Innovation Center for Efficient and Green Production of Agriculture in Mountainous Areas of Zhejiang Province, College of Horticulture Science, Zhejiang Agriculture & Forestry University, Hangzhou, China

**Keywords:** LINE-1, Transposable element, retrotransposition, *BrCER2*, cuticular wax biosynthesis, *Brassica rapa* L. ssp. *pekinensis*

## Abstract

Glossiness is an important quality-related trait of Chinese cabbage, which is a leafy vegetable crop in the family Brassicaceae. The glossy trait is caused by abnormal cuticular wax accumulation. In this study, on the basis of a bulked segregant analysis coupled with next-generation sequencing (BSA-seq) and fine-mapping, the most likely candidate gene responsible for the glossy phenotype of Chinese cabbage was identified. It was subsequently named *Brcer2* because it is homologous to *AtCER2* (At4g24510). A bioinformatics analysis indicated a long interspersed nuclear element 1 (LINE-1) transposable element (named *BrLINE1-RUP*) was inserted into the first exon of *Brcer2* in HN19-G via an insertion-mediated deletion mechanism, which introduced a premature termination codon. Gene expression analysis showed that the InDel mutation of *BrCER2* reduced the transcriptional expression levels of *Brcer2* in HN19-G. An analysis of cuticular waxes suggested that a loss-of-function mutation to *BrCER2* in Chinese cabbage leads to a severe decrease in the abundance of very-long-chain-fatty-acids (> C28), resulting in the production of a cauline leaf, inflorescence stem, flower, and pistil with a glossy phenotype. These findings imply the insertion of the LINE-1 transposable element *BrLINE1-RUP* into *BrCER2* can modulate the waxy traits of Chinese cabbage plants.

## Introduction

Chinese cabbage (*Brassica rapa* L. ssp. *pekinensis*) is an important vegetable crop in the family Brassicaceae that is widely cultivated in northeastern Asia. Leaf and stalk glossiness is a commercially important quality-related trait among *Brassica* species, including *Brassica rapa* and *Brassica oleracea*. Remarkably, compared with waxy leaf and stalk, glossy leaf and stalk are more attractive to consumers. Previous studies showed that the glossy phenotype is due to defective cuticular wax biosynthesis (Liu et al., 2018; Ji et al., 2021; Yang et al., 2022a; Yang et al., 2022b). Cuticular waxes are classified as intracuticular waxes and epicuticular waxes based on their location. Intracuticular waxes are deposited within the cutin matrix, while epicuticular waxes cover on top of intracuticular wax (Haslam et al., 2012). The structural and chemical characteristics of cuticular wax vary greatly among plant species, tissues, genotypes, and developmental stages (Arya et al., 2021). Cuticular waxes are formed by a complex mixture of C20–C40 very-long-chain-fatty-acids (VLCFAs) and their derivatives, including alkanes, ketones, aldehydes, primary and secondary alcohols, and esters (Samuels et al., 2008; Isaacson et al., 2009). Besides, triterpenoids are also present in cuticular waxes and are main components of cuticular waxes in some species such as olives and grapes (Diarte et al., 2019; Arand et al., 2021).

In *Arabidopsis thaliana*, the related genes and enzymes involved in VLCFA biosynthesis have been thoroughly characterized. The C16 and C18 fatty acids are synthesized in the plastids of epidermal cells and then elongated to VLCFAs in the endoplasmic reticulum by fatty acid elongase complexes consisting of the following four enzymes: β-ketoacyl-CoA synthase, β-ketoacyl-CoA reductase, β-hydroxyacyl-CoA dehydratase, and β-enoyl-CoA reductase (Millar and Kunst, 1997; Samuels et al., 2008). Two functionally redundant genes (*KCS2* and *KCS20*) encode the proteins responsible for the elongation of C20 fatty acids to C22 fatty acids (Lee et al., 2009). Additionally, KCS9 mediates the elongation of C22 fatty acids to C24 fatty acids, whereas KCS1 is required for the elongation of C24 VLCFAs (Todd et al., 1999; Kim et al., 2013). The silencing of *KCS1* expression decreases the C26–C30 wax alcohol and aldehyde levels by up to 80% (Todd et al., 1999). Moreover, KCS5/CER60 and KCS6/CER6 play redundant roles during the production of the C26–C28 fatty acids involved in wax biosynthesis (Millar et al., 1999; Fiebig et al., 2000; Trenkamp et al., 2004). Two BAHD acyltransferases (CER2 and CER26) contribute to C28 and C30 fatty acid elongation (Haslam et al., 2012; Pascal et al., 2013).

Bulked segregant analysis (BSA) is an efficient approach to rapidly mine genes responsible for mutant phenotypes (Liu et al., 2012). Main procedure of BSA includes selecting two types of segregating individuals with extremely opposing phenotypes, pooling respectively the DNA of all individuals to form two bulks of DNA pools, and identifying genetic markers strongly associated with targeted genes (Giovannoni et al., 1991; Zou et al., 2016). The recent and rapid advance of next-generation sequencing (NGS) technology promotes the development and application of BSA-seq technology (BSA coupled with whole-genome sequencing), which has been extensively applied to identify trait-related genes in plants (Zou et al., 2016). In Chinese cabbage, three waxy genes (*BrWAX1*, *BrWAX2*, and *BrWAX3*) have been mapped and cloned by BSA-seq and fine mapping. They were involved in epidermal wax biosynthesis and responsive for waxy phenotype (Zhang et al., 2013; Liu et al., 2021; Yang et al., 2022a; Yang et al., 2022b).

Transposable elements (TEs) are major drivers of plant genome evolution. In plants, TEs facilitate the duplication or deletion of genes, modulate gene expression or function, and combine genes from different genomic locations (i.e., gene fusions) (Bennetzen, 2000; Krasileva, 2019). More specifically, TEs are mobile DNA segments that are capable of replicating and changing positions in the genome. They are generally divided into two categories (DNA transposons and retrotransposons) according to how they are mobilized (Wicker et al., 2007). Briefly, DNA transposons move via a cut-and-paste mechanism, whereas retrotransposons move via a copy-and-paste process that involves the duplication and incorporation of a sequence into a new genomic location through an RNA intermediate (Kim et al., 2012). Non-long terminal repeat (LTR) retrotransposons include long interspersed nuclear elements (LINEs) and short interspersed nuclear elements (SINEs). Of these two elements, LINEs usually comprise two open reading frames (ORFs). The first ORF (i.e., ORF1) encodes an RNA-binding protein, whereas ORF2 encodes a protein that has endonuclease and reverse transcriptase activities. Additionally, LINEs contain a poly(A) stretch, poly(T) stretch, or simple sequence motifs at the 3′ end and are flanked by a sequence modified by a target site duplication (TSD) (Wicker et al., 2007).

In this study, the VLCFA biosynthesis-related gene (*BrCER2*) on chromosome A01 of Chinese cabbage was identified by BSA-seq and fine mapping. A loss-of-function mutation to *BrCER2* caused the waxy phenotype of the cauline leaf, inflorescence stem, flower, and pistil to change to a glossy phenotype. A partial LINE-1 retrotransposon (*BrLINE1-RUP*) sequence inserted itself into the first exon of *BrCER2* in an insertion-mediated deletion manner, resulting in a mutated *BrCER2* gene. Our findings have clarified the molecular mechanism underlying the *BrCER2*-mediated regulation of the biosynthesis of the VLCFAs in the cuticular waxes of Chinese cabbage. Specifically, we confirmed that *BrLINE1-RUP* is an active LINE-1 retrotransposon and revealed that its insertion into the *BrCER2* exon is the cause of the glossy phenotype of Chinese cabbage.

## Materials and methods

### Plant materials

**Figure 1:**
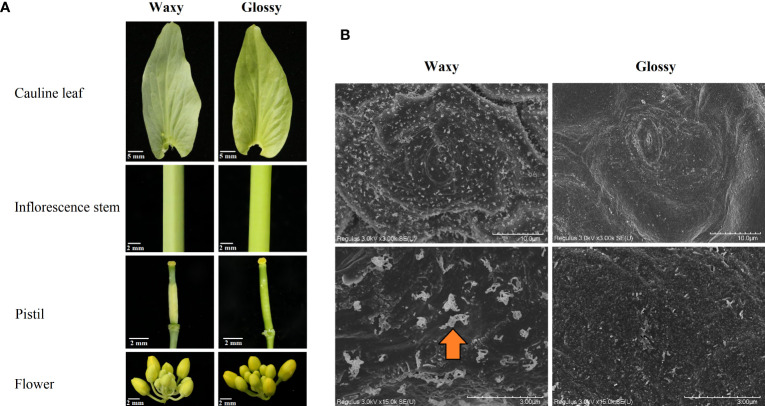
Phenotype of HN19-G (glossy) and QM19 (waxy) plants **(A)**. Scanning electron microscopy images of the cauline leaves from HN19-G (glossy) and QM19 (waxy) plants **(B)**. The arrowhead indicates cuticular wax crystals.

Lines QM19 and HN19-G of Chinese cabbage (*B. rapa* L. ssp. *pekinensis*) respectively have a traditional waxy phenotype and a glossy phenotype (cauline leaf, inflorescence stem, flower, and pistil) (**Figure 1A**). An F_2_ population (896 plants) derived from the QM19 × HN19-G hybridization was used for the BSA-seq and fine-mapping of the *Brwax* gene. The chi-square test (IBM SPSS Statistics 26.0) was used to determine the fit of the segregation ratio of the F_2_ generation to the expected ratio.

### Scanning electron microscopy analysis

Two fresh cauline leaves (having similar size) obtained from HN19-G and QM19 plants at the flowering stage were fixed for 2-4 hours by 2.5% glutaraldehyde fixing solution and rinsed 3 times with 0.1 M phosphate buffer (pH 7.0), subsequently fixed for 1-3 hours with 1% osmic acid · 0.1 M phosphate buffer (pH 7.0) and washed 3 times by 0.1 M phosphate buffer (pH 7.0). The samples were dehydrated by 50%, 70%, 80%, 90%, 95%, and 100% alcohol (two times) for 15 minutes each time and permeated with 100% alcohol: isoamyl acetate (1:1) for 30 minutes and permeated overnight by pure isoamyl acetate. The samples were dried and transferred to a preparation chamber under vacuum for coating. The photographs of the adaxial and abaxial surface of the sample were taken using scanning electron microscopy (SEM) system (Hitachi 8100, Tokyo, Japan).

### Bulked segregant analysis and next-generation sequencing

The BSA-seq analysis was conducted using two pooled samples of 50 glossy phenotype (G-bulk) and 50 waxy phenotype (W-bulk) F_2_ segregants as well as the two parental lines (HN19-G and QM19). The Illumina Nova 6000 platform was used to generate 150-base paired-end reads for the four pools by Biomarker technologies Co., Ltd. (Beijing, China). The raw data was deposited in the Sequence Read Archive (SRA) in NCBI as PRJNA967584. The *B. rapa* reference genome v3.0 and GATK were used to identify high-quality single nucleotide polymorphisms (SNPs) and insertions/deletions (InDels). The SNP-index and InDel-index were calculated at each position for the G-bulk and W-bulk. The ΔSNP-index of each SNP position was calculated by subtracting the SNP-index of the G-bulk from the SNP-index of the W-bulk (Fekih et al., 2013; Hill et al., 2013). The ΔInDel-index of each InDel position was similarly calculated. Significant linkage disequilibrium was used to identify the candidate region for the glossy trait (correlation threshold = 0.54) (BIOMARKER; Beijing, China). The intersection of the linked regions (ΔSNP-index and ΔInDel-index) was selected as the final candidate linked region (**Table S1**).

### Fine-mapping of *Brwax*

Polymorphic primer sets (**Table S2**) were used to analyze the genotype of the plants in the F_2_ population with glossy and waxy phenotypes. The recombination events were assayed to delimit the region containing *Brwax*.

The PCR products produced using the primers for the M81 marker were examined by 1% agarose gel electrophoresis. The amplified fragments differed between the glossy and waxy F_2_ plants derived from the HN19-G × QM19 hybridization. One fragment (198 bp) was amplified for the glossy plants (*BrwaxBrwax*). In contrast, one fragment (108 bp) and two fragments (198/108 bp) were amplified for the homozygous waxy plants (*BrWAXBrWAX*) and the heterozygous waxy plants (*BrWAXBrwax*), respectively.

### Candidate gene prediction

The sequences and chromosome position information of these genes within the target region were obtained from *B. rapa* genome v3.0 deposited in the Brassica database (http://brassicadb.cn/). Function of each gene was anotated based on the corresponding *Arabidopsis* homolog, deduced by the BLAST analysis from the National Center for Biotechnology Information (http://blast.ncbi.nlm.nih.gov/Blast.cgi). The sequences of the candidate genes of HN19-G and QM19 were acquired from resequencing data and were aligned by ClustalX software. The InDel fragment in HN19-G was verified by PCR with the M81 marker.

### Identification and characterization of the inserted fragment in *Brcer2*

The *BrCER2* sequence in QM19 and the *Brcer2* sequence in HN19-G were analyzed using the raw resequencing data for QM19 and HN19-G. The 130-bp inserted fragment in *Brcer2* of HN19-G was retrieved from the *B. rapa* genome v3.0 in BRAD (http://brassicadb.cn/#/) to determine its origin (Zhang et al., 2018; Priyam et al., 2019). The potential TE was further analyzed and grouped according to the *B. rapa* genome v3.0 TE database in BRAD (http://brassicadb.cn/#/) (Zhang et al., 2018). Target site duplications were analyzed using REPuter in BiBiserv2 (https://bibiserv.cebitec.uni-bielefeld.de/sessionTimeout.jsf) (Kurtz et al., 2001). The TE ORFs were analyzed by aligning *BrLINE1-RUP* with other annotated LINE-1 sequences (http://repeatmasker.org). The position of *BrLINE1-RUP* in the *B. rapa* genome v3.0 was determined using JBrowse (http://brassicadb.cn/#/) (Zhang et al., 2018). A PCR amplification was performed using specific primer pairs (**Table S2**) to clarify the mechanism mediating the transposition of *BrLINE1-RUP*. BioEdit was used to analyze *BrLINE1-RUP* in the *B. oleracea*, *B. rapa*, *A. thaliana*, *Raphanus sativus*, and *Brassica nigra* genomes as well as in 18 other representative *B. rapa* genomes (http://brassicadb.cn/#/) (Cai et al., 2021).

### Gas chromatography and mass spectrometry (GC-MS)

The G-bulk and W-bulk were respectively prepared by mixing equal amounts of entire cauline leaves (having similar size, 5-7 cm^2^) at the flowering stage from 20 glossy (*BrwaxBrwax*) and 20 waxy F_2_ individuals (*BrWAXBrWAX : BrWAXBrwax=*7:13). Three biological replicates of the W-bulk and G-bulk were assessed. The pictures of cauline leaves were taken to determine surface area of cauline leaf using ImageJ. The total cuticular waxes were collected by soaking the leaves in chloroform for 30 s and 2 µL tetracosane (10 mg/mL) (C24, SUPERLCO, Sigma) was added as an internal standard. The chloroform was evaporated under a stream of gaseous nitrogen. The sample was dissolved with 100μL hexane, subsequently incubated for 60 min at 70°C after adding 100 μL-bis(trimethylsilyl)fuoroacetamide (BSTFA, SUPERLCO, sigma). These derivatized samples were analyzed using a GC-MS system (Agilent 7890B-5977B GC–MS) at Shanghai Jiao Tong University. The initial temperature of 50°C was held for 2 min, increased at 20°C/min to 200°C, increased again at 3°C/min to 310°C, and held for 10 min at 310°C. Compounds were quantified according to the flame ionization detector peak areas and the internal standard (C24 alkane) (Yang et al., 2022a; Yang et al., 2022b). Cuticular wax content was calculated across three biological replications per composition and indicated as mean + standard deviation (SD) (n=3). Statistical analysis was performed using Student’s *t*-test.

### Gene expression analysis

To analyze *BrCER2* and *Brcer2* expression in a common genetic background, we constructed the HN19-G near isogenic line, which was subsequently named HN19-W. The detailed scheme for HN19-W development was described in **Figure S1**. Details regarding the primer sets are provided in **Table S2**. Relative gene expression levels in the root, rosette leaf, cauline leaf, inflorescence stem, flower, and pistil were determined using the ABI StepOne™ Real-Time PCR System (Applied Biosystems) and the 2^−ΔΔCt^ method. Relative expression levels were normalized first against the *BrACT7* transcript level (i.e., internal control) and then against the expression level in the flower of HN19-W. The relative fold-change in the expression of each gene was calculated across all biological and technical replicates. Relative gene expression levels are presented herein as the mean + standard deviation.

### RNA-seq analysis of the near isogenic line

The cauline leaves of HN19-G and HN19-W were sampled at the same developmental stage. Total RNA was extracted and sequenced by the Illumina Nova 6000 platform (BIOMARKER; Beijing, China). The raw data, which was composed of 150-base paired-end reads, was deposited in the Sequence Read Archive (SRA) in NCBI as PRJNA968036. The clean reads for each sample were aligned to the *B. rapa* genome v3.0 (http://brassicadb.cn/#/Download/). Gene expression levels were determined in terms of FPKM values. Differentially expressed genes (DEGs) (i.e., fold-change ≥ 2 and false discovery rate < 0.01) were identified.

## Results

### The glossy phenotype of HN19-G is controlled by a recessive nuclear gene

The examination of the cauline leaf, inflorescence stem, flower, and pistil indicated QM19 has the traditional waxy phenotype, which is in contrast to the glossy phenotype of HN19-G (**Figure 1A**). The SEM analysis indicated that unlike QM19, HN19-G has less cuticular wax crystals, which are composed of VLCFAs and their derivatives (**Figure 1B**). These findings suggested that VLCFA biosynthesis was affected in HN19-G. All F_1_ plants, which were derived from a cross between a glossy parent (HN19-G) and a waxy parent (QM19), had a waxy phenotype. Of the 896 F_2_ plants, 675 had a waxy phenotype and 221 had a glossy phenotype. The F_2_ segregation ratio corresponded to the expected Mendelian ratio of 3:1 (χ^2^ < χ^2^_[df = 1, P = 0.05]_) according to the χ^2^ test (**Table S3**). Accordingly, the glossy phenotype of HN19-G is likely controlled by a recessive nuclear gene (i.e., *Brwax*).

### Preliminary mapping of the *Brwax* locus

**Figure 2:**
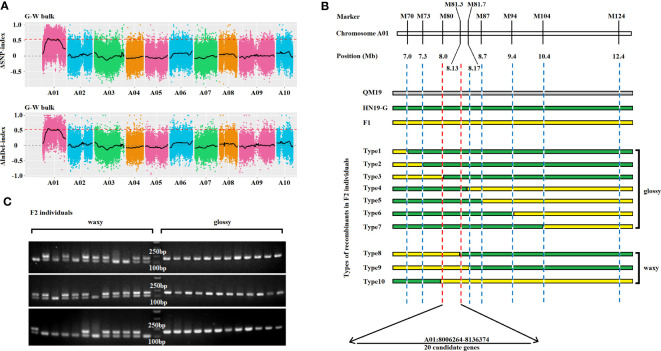
Gene mapping and candidate gene analysis for the glossy phenotype gene *Brwax*. **(A)** Preliminary mapping of *Brwax* on the basis of ΔSNP-index and ΔInDel-index (threshold value = 0.54), which were calculated at 4-Mb intervals with a 10-kb sliding window. **(B)** Fine-mapping of *Brwax* according to recombination events using molecular markers. **(C)** Comparative analysis of the genotypes and phenotypes of F_2_ plants using the M81 marker.

To preliminarily map the *Brwax* gene, a BSA-seq analysis was performed using the waxy (W-bulk) and glossy (G-bulk) F_2_ segregants and the two parental lines (HN19-W and QM19). In total, 65229700 and 82759172 clean reads were generated from the G-bulk and W-bulk, respectively. The Q30 (those reads with an average quality score >30) was >91%, indicating that the sequencing results was highly accurate (**Table S4**). Using the *B.* genome v3.0 as a reference, average sequencing depth of G-bulk and W-bulk was 53× and 67×, respectively (**Table S4**). Moreover, *B. rapa* genome v3.0 was used to identify SNPs and InDels in the W-bulk and the G-bulk. The ΔSNP-index of each SNP position and the ΔInDel-index of each InDel position were calculated via a sliding window analysis. The correlation threshold was set as 0.54. The final target regions were located on chromosome A01: 6,210,000–8,680,000 bp and 19,120,000–19,170,000 bp (**Figure 2A**; **Table S1**).

### Fine mapping of the *Brwax* locus

To narrow the target region, 869 F_2_ plants were selected as the fine-mapping population. The primer pairs used for detecting recombination events revealed that *Brwax* was flanked by M70, M73, M79, and M80 on one side and M81.3, M81.7, M87, M94, M104, and M124 on the other side. The *Brwax* gene was delimited to a 130.1-kb region (A01: 8,006,264–8,136,374) flanked by the M80 and M81.3 markers (**Figure 2B**). The M81 marker was used to compare the F_2_ plant genotypes and phenotypes. This marker co-segregated with *Brwax* (**Figure 2C**).

### Candidate gene analysis

By screening the *B. rapa* genome v3.0, we identified and annotated 20 genes in the target region (**Table 1**). *BraA01g015290.3C* was identified as the most likely gene responsible for the glossy phenotype. Because *BraA01g015290.3C* was revealed as a homolog of *AtCER2* (At4g24510) in *A. thaliana*, it was named *BrCER2*. In *A. thaliana*, *AtCER2* is involved in the biosynthesis of cuticular wax and contributes to VLCFA biosynthesis. Specifically, its expression is required for C28 fatty acid elongation in the stem (Haslam et al., 2012; Pascal et al., 2013).

**Table 1:** Candidate genes in the region.

Gene ID	Gene Position on A01	*Arabidopsis* Homolog	Gene function
*BraA01g015140.3C*	(-):8006268.8008413	At5g49945	Uncharacterized protein At5g49945
*BraA01g015150.3C*	(+):8017453.8019603	AT4G24340	Phosphorylase superfamily protein
*BraA01g015160.3C*	(+):8020433.8023137	AT4G24350	Phosphorylase superfamily protein
*BraA01g015170.3C*	(-):8023924.8024391	AT4G24370	uncharacterized protein
*BraA01g015180.3C*	(+):8025846.8026894	AT4G24380	Galectin domain-containing protein
*BraA01g015190.3C*	(-):8027085.8029550	At4g24390	F-box protein FBX14
*BraA01g015200.3C*	(+):8034080.8036979	At4g24400	CBL-interacting serine/threonine-protein kinase 8
*BraA01g015210.3C*	(+):8058021.8061322	AT4G24430	rhamnogalacturonan endolyase
*BraA01g015220.3C*	(+):8067384.8068634	At4g24440	Transcription initiation factor IIA subunit 2
*BraA01g015230.3C*	(+):8069555.8076148	At4g24450	Alpha-glucan water dikinase 2
*BraA01g015240.3C*	(+):8076897.8079368	At4g24460	Protein CLT2, chloroplastic
*BraA01g015250.3C*	(+):8083117.8085090	At4g24470	GATA transcription factor 25
*BraA01g015260.3C*	(+):8095886.8100171	AT4G24480	Dolichyl-diphosphooligosaccharide–protein glycosyltransferase subunit 2
*BraA01g015270.3C*	(-):8100701.8104059	At4g24490	Geranylgeranyl transferase type-2 subunit alpha 1
*BraA01g015280.3C*	(+):8104966.8106332	At4g24500	Protein SICKLE
***BraA01g015290.3C***	**(+):8107040.8110388**	**At4g24510**	**HXXXD-type acyl-transferase family protein for C28 to C30 fatty acid elongation**
*BraA01g015300.3C*	(+):8111194.8115045	At4g24520	NADPH–cytochrome P450 reductase 1
*BraA01g015310.3C*	(-):8115672.8119576	At4g24520	NADPH–cytochrome P450 reductase 1
*BraA01g015320.3C*	(-):8120215.8126066	At4g24530	O-fucosyltransferase 31
*BraA01g015330.3C*	(+):8133677.8136665	At4g24550	AP-4 complex subunit mu

The most likely candidate gene was shown in bold.

**Figure 3:**
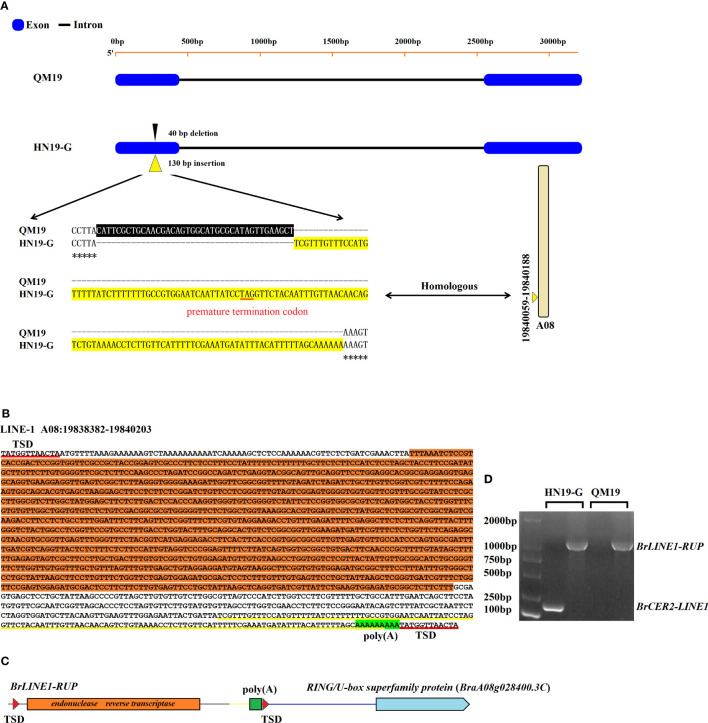
The mutation in *Brcer2* of HN19-G was due to the retrotransposition of *BrLINE1-RUP.* **(A)** A 40-bp deletion (black background) and a 130-bp insertion (yellow background) were detected in the first exon of *Brcer2* in HN19-G. The sequence of the 130-bp insertion was identical to a sequence on chromosome A08 (19,840,059–19,840,188). **(B)** Sequence and structure of a LINE-1 retrotransposon on chromosome A08 (19,838,382–19,840,203). The ORF2 sequence of LINE-1 (endonuclease and reverse transcriptase) is indicated with an orange background. The 130-bp sequence of LINE-1 that was identical to that in *Brcer2* of HN19-G is marked by a yellow underline. **(C)** Schematic diagram of *BrLINE1-RUP.* **(D)** Products of the PCR amplification of *BrLINE1-RUP* and *Brcer2-LINE1* in HN19-G and QM19. TSD, target site duplication.

The sequencing of *Brcer2* in HN19-G and *BrCER2* in QM19 indicated that *BrCER2* in the waxy parent QM19 comprises 3,349 bp and contains two exons and one intron (**Figure 3A**). The *BrCER2* coding sequence in QM19 is 1,254 bp long and is similar to *AtCER2* (At4g24510) in *A. thaliana* (80.7% sequence identity). However, *Brcer2* in the glossy parent HN19-G consists of 3,438 bp, which includes a 1,344-bp coding sequence. A 40-bp deletion and a 130-bp insertion were identified in the first exon of *Brcer2* in HN19-G (**Figure 3A**). A premature termination codon was detected in the 130-bp insertion, resulting in the expression of a non-functional truncated protein (**Figure 3A**). A functional marker (M81) for *BrCER2* and *Brcer2* co-segregated with *Brwax* (**Figure 2C**).

### Identification and characterization of a newly LINE-1 TE (*BrLINE1-RUP*)

The 130-bp insertion was used as the query for a BLAST search of the *B. rapa* genome v3.0, which detected 184 homologous copies dispersed on all 10 chromosomes in the *B. rapa* genome (**Table S5**). This finding suggested that the 130-bp insertion in *Brcer2* of HN19-G was probably from a TE that replicated itself within genomes. The 130-bp insertion was potentially derived from the transposition of another TE. Among the 184 homologous fragments, the unique sequence on chromosome A08 (19,840,059–19,840,188) was identical to the 130-bp insertion in *Brcer2* (**Figure 3A**). Hence, we speculated that the fragment on chromosome A08 may be a TE that replicated itself and produced the mutated *Brcer2* in HN19-G. To verify this possibility, we analyzed a 10-kb sequence containing the fragment on chromosome A08 (19,840,059–19,840,188). The fragment was flanked by a TSD site, which is a characteristic of transposons. The potential transposon contained homologous sequences encoding an endonuclease and reverse transcriptase and a poly(A) sequence (**Figure 3B**), which is required for the target-primed reverse transcription of the LINE-1 element. It was localized to the promoter of a gene encoding a RING/U-box superfamily protein (*RUP*; *BraA08g028400.3C*) (**Figure 3C**). According to its structure and location, the TE was considered to be a LINE-1 retrotransposon and named *BrLINE1-RUP*. Thus, the 130-bp insertion on chromosome A01 (*Brcer2-LINE1*) was derived from the transposition of *BrLINE1-RUP* on chromosome A08.

To identify the mechanism mediating the transposition of *BrLINE1-RUP*, PCR amplifications were performed using primer pairs designed for detecting *BrLINE1-RUP* on chromosome A08 and *Brcer2-LINE1* on chromosome A01. The PCR results indicated *BrLINE1-RUP* is present in QM19, whereas *Brcer2-LINE1* is not (control). However, two PCR products (*Brcer2-LINE1* on chromosome A01 and *BrLINE1-RUP* on chromosome A08) were detected for HN19-G, indicating *BrLINE1-RUP* remained in its original position after the transposition of *BrLINE1-RUP* into *Brcer2* (**Figure 3D**). Because of this copy-and-paste mechanism, *BrLINE1-RUP* is probably a retrotransposon. The transposition of *BrLINE1-RUP* into *Brcer2* on chromosome A01 did not result in TSDs flanking the inserted fragment, but *Brcer2* was missing a 40-bp fragment. In previous studies, researchers detected LINE-1 insertion-mediated deletions (L1IMDs) and suggested the LINE-1 element size may be correlated with the size of the corresponding deleted fragment (Han et al., 2005). The results of the current study also indicated that the retrotransposition of *BrLINE1-RUP* involved an insertion-mediated deletion, resulting in a lack of TSDs in *Brcer2-LINE1* (**Figure 3A**).

**Figure 4:**
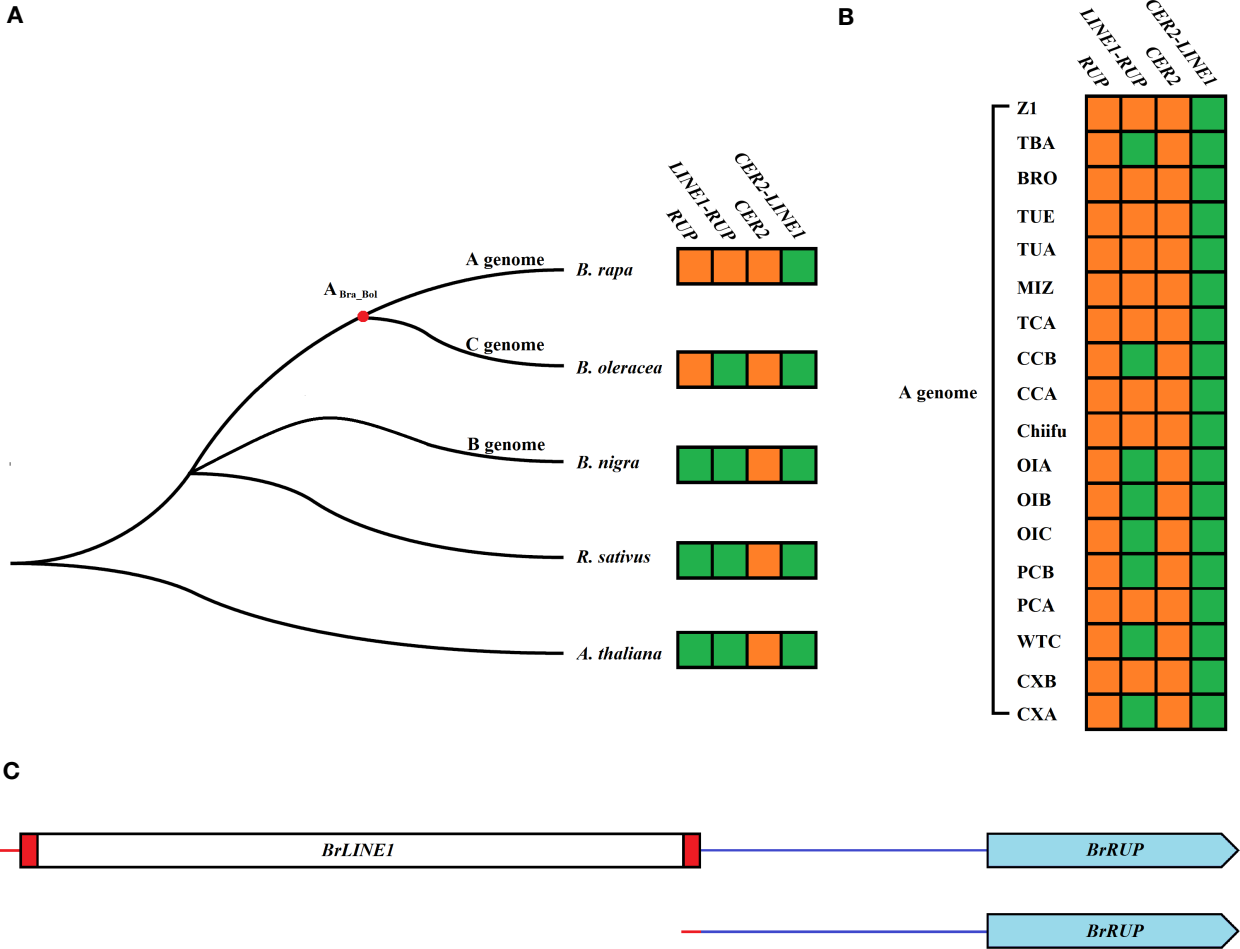
*LINE1-RUP* and *CER2-LINE1* in the genomes of five *Brassica* species **(A)** and 18 representative *B. rapa* genomes **(B)**. The phylogenetic relationships among Brassicaceae crops were obtained from a previous study (Cheng et al., 2017). Orange and green square blocks indicate presence and absence, respectively. **(C)** Structural comparison of *BrLINE1-RUP* (*LINE-1* insertion in *BrRUP*) and *BrRUP* (no transposition) in *B. rapa*.

To investigate the origins of *LINE1-RUP* and *cer2-LINE1*, *Brassica* species were screened for *RUP*, *LINE1-RUP*, *cer2*, and *cer2-LINE1*. Although *RUP* genes were detected in *B. oleracea* and *B. rapa*, they were undetectable in *A. thaliana*, *R. sativus*, and *B. nigra.* In contrast, *LINE1-RUP* was exclusive to *B. rapa* (**Figure 4A**). Both *RUP* and *LINE1-RUP* were also detected in 18 representative *B. rapa* genomes, implying that *RUP* is present in all 18 *B. rapa* genomes. However, 10 *B. rapa* genomes contained *BrLINE1-RUP*, whereas eight *B. rapa* genomes only contained *BrRUP* (**Figure 4B**; **Table S6**). The comparison of the *BrRUP* and *BrLINE1-RUP* sequences indicated the *LINE-1* transposition into the *RUP* promoter led to the production of a new *LINE1-RUP* TE in the A genome (**Figures 4C**, **S2**). A PCR analysis of 56 *B. rapa* L. ssp. *pekinensis* lines identified seven lines carrying *BrLINE1-RUP* in their genome (**Figure S3**). Furthermore, *CER2* genes were detected in *A. thaliana*, *R. sativus*, *B. nigra*, *B. oleracea*, and *B. rapa*, but *cer2-LINE1* was not detected in the genomes of these species. Similarly, *cer2-LINE1* was also absent in 18 representative *B. rapa* genomes (**Figure 4B**).

### Cuticular wax analysis of W-bulk and G-bulk

**Figure 5:**
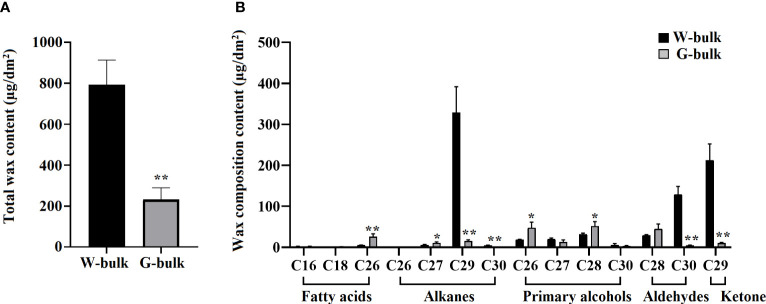
Cuticular wax composition in the cauline leaves of the W-bulk and G-bulk. **(A)** Total wax contents in the W-bulk and G-bulk, calculated as average values for three biological replicates. **(B)** Wax compositions in the W-bulk and G-bulk, measured as average values for three biological replicates. Error bars indicate SD (n = 3); **P<0.01 (Student’s *t* test). *0.01<P<0.05 (Student’s *t* test).

To elucidate the mechanism underlying the glossy trait of HN19-G, cuticular wax from the W-bulk and G-bulk was collected for the GC-MS analysis. The average total wax content was considerably higher for the waxy leaves (792.23 µg/dm^2^ surface area) than for the glossy leaves (231.85 µg/dm^2^ surface area). Hence, the wax content was 71% lower for the G-bulk than for the W-bulk (**Figure 5**; **Table S7**). The wax composition analysis revealed that the major waxes in the W-bulk were C29 alkane, C29 ketone, and C30 aldehyde, whereas they were C26 and C28 primary alcohols, C28 aldehyde, and C26 fatty acid in the G-bulk. The C29 alkane, C30 aldehyde, and C29 ketone contents in the leaves of the G-bulk were respectively only 4.7%, 3.5%, and 4.8% of the corresponding levels in the leaves of the W-bulk (**Figure 5**; **Table S7**). However, the C26 fatty acid, C27 alkane, C28 primary alcohol, and C28 aldehyde contents were higher in the G-bulk than in the W-bulk (**Figure 5**; **Table S7**). Overall, the abundance of the long-chain waxes (> C28) decreased substantially in the glossy plants. Conversely, the VLCFA (< C28) contents were greater in the glossy plants than in the waxy plants. These findings suggested that *BrCER2* encodes the protein responsible for C28 fatty acid elongation, similar to *AtCER2* in *A. thaliana.*

### Comparative analysis of *BrCER2* and *Brcer2* expression levels

**Figure 6:**
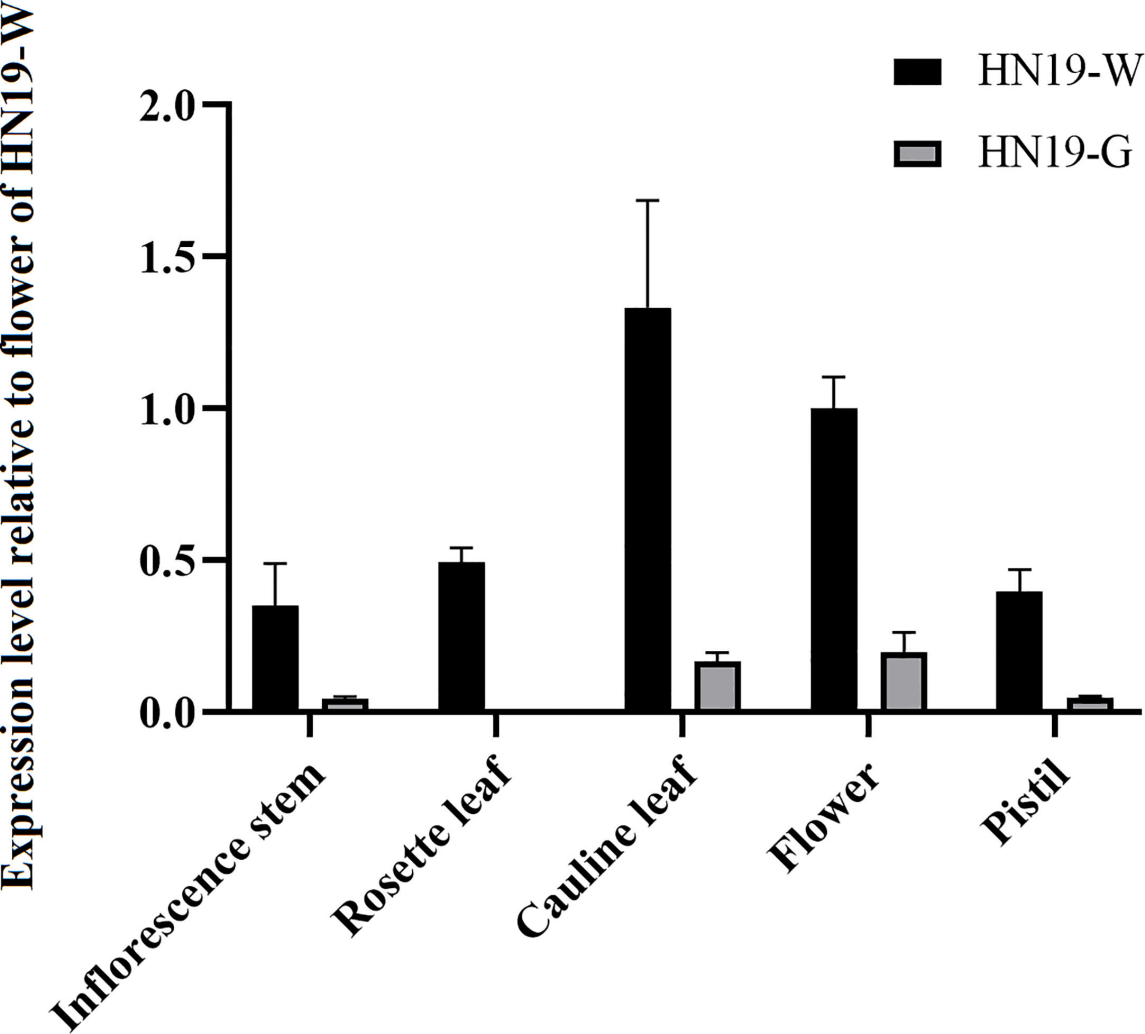
Expression of *BrCER2/Brcer2* transcripts in the inflorescence stem, rosette leaf, cauline leaf, flower, and pistil. Error bars indicate SD (n = 3).

The *BrCER2* and *Brcer2* expression levels were analyzed by completing a quantitative real-time polymerase chain reaction assay (qRT-PCR). In HN19-W, *BrCER2* was most highly expressed in the cauline leaf, followed by the flower, rosette leaf, pistil, and inflorescence stem. In HN19-G, *Brcer2* expression was highest in the flower, cauline leaf, pistil, and inflorescence stem, followed by the rosette leaf (**Figure 6**). The 40-bp deletion and 130-bp insertion produced a premature termination codon in *Brcer2* in HN19-G (**Figure 3A**). Premature termination codons in mRNA generally lead to decreased mRNA abundance due to nonsense-mediated decay, which is a post-transcriptional mechanism for regulating gene expression. To analyze the effect of InDel on *Brcer2* expression, a comparative expression analysis was performed. The results showed that the *Brcer2* expression level in the cauline leaf, flower, rosette leaf, pistil and inflorescence stem of HN19-G was clearly lower than the *BrCER2* expression level in the cauline leaf, flower, rosette leaf, pistil and inflorescence stem of HN19-W (**Figure 6**).

### Analysis of the waxy and glossy cauline leaf transcriptomes

A comparative transcriptome analysis of the waxy cauline leaf of HN19-W and the glossy cauline leaf of HN19-G was performed to screen for DEGs and regulatory networks involved in wax biosynthesis. Approximately 121.9 million clean reads were produced for the six samples, ranging from 19.1 to 21.3 million clean reads per library (**Table S8**). Among the clean reads, 87.69–90.47% were uniquely mapped to *B. rapa* genome v3.0 (**Table S8**). There were 301 DEGs (fold change ≥2 and false discovery rate <0.01), among which 129 genes were upregulated and 172 genes were downregulated in the glossy cauline leaf compared with in the waxy cauline leaf (**Table S9**). RNA-seq results were verified by qRT-PCR analysis (**Figure S4**).

**Figure 7:**
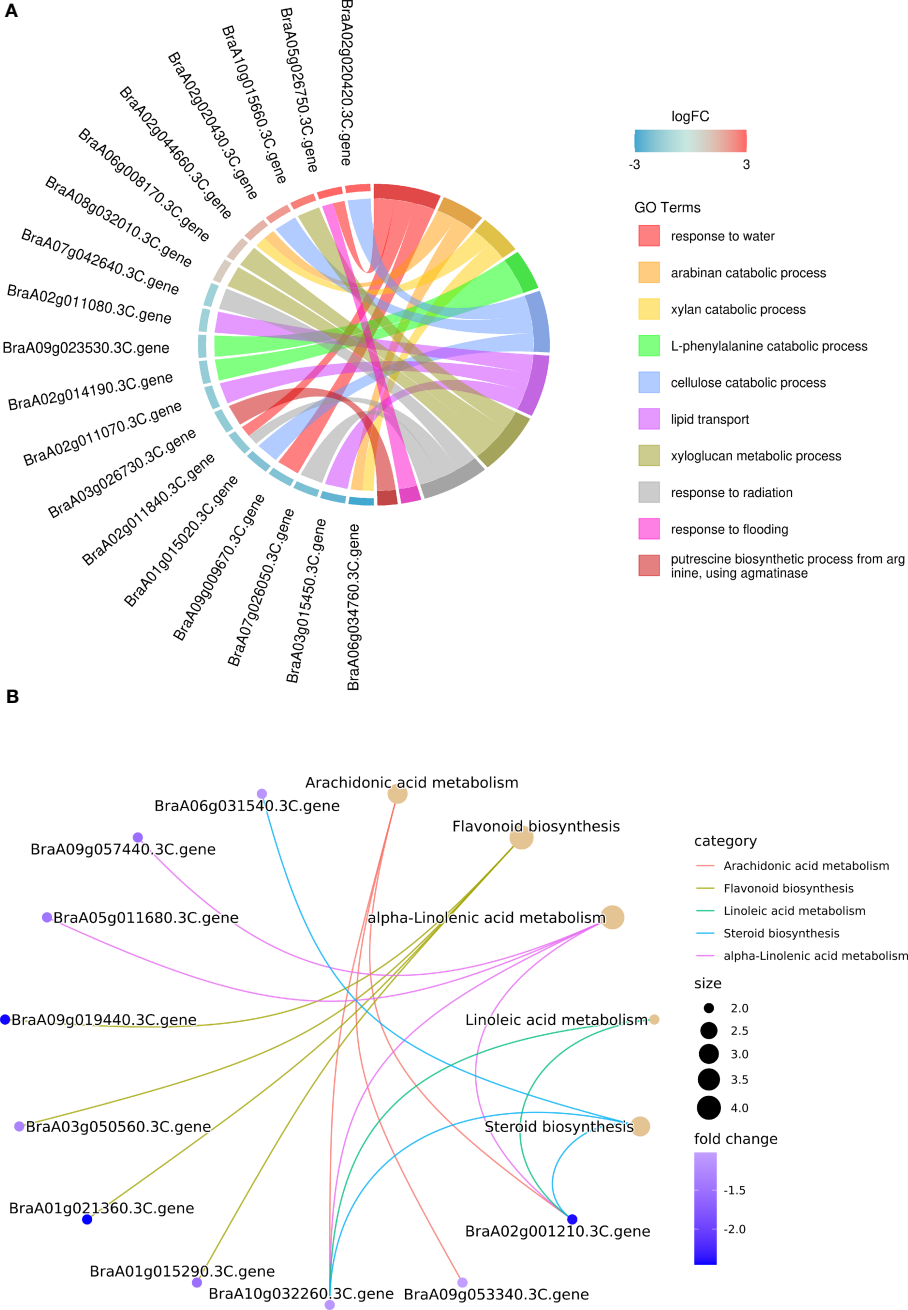
**(A)** GO enrichment analysis of DEGs for biological processes. The DEGs were annotated to top 10 GO terms (with smallest Q-value). **(B)** KEGG pathway enrichment analysis of DEGs for biological processes. Top 5 enriched pathways (with smallest Q-value) were shown with brown dots, whose size represents the number of DEGs enriched in the corresponding pathway.

Gene Ontology (GO) enrichment analysis (biological process) showed that lipid transport processes were enriched. *BraA02g011070.3C*, *BraA02g011080.3C* and *BraA03g015450.3C*, which encode non-specific lipid-transfer proteins, were significantly downregulated in the glossy cauline leaf (**Figure 7A**). Previous studies indicated that non-specific lipid-transfer proteins may play a role in wax or cutin deposition in epidermal cells (Liu et al., 2014; Deeken et al., 2016).

The main enriched Kyoto Encyclopedia of Genes and Genomes pathways (KEGG) among these DEGs were arachidonic acid metabolism, flavonoid biosynthesis, alpha-linolenic acid metabolism, linoleic acid metabolism, and steroid biosynthesis, and all of them were downregulated in the glossy cauline leaf of HN19-G (**Figure 7B**). In plant, VLCFA can be converted into other lipids mediated by very long-chain acyl-CoAs, which were produced by fatty acid elongation complexes (Batsale et al., 2021). We deduced that down-regulation of these genes may reduce the conversion from VLCFA into other lipids to compensate for wax loss in glossy plants.

In cutin, suberin and wax biosynthesis, *BraA02g026450.3C* homologous to *CYP86A2* of *Arabidopsis thaliana* (At4g00360) were significantly upregulated in the glossy cauline leaf. In *Arabidopsis thaliana*, CYP86A2 is a cytochrome P450 monooxygenase catalyzing fatty acid oxidation. The cutin content is reduced to 30% in *cyp86a2* mutants, indicating that CYP86A2 plays a major role in the biosynthesis of extracellular lipids (Xiao et al., 2004). However, *BraA01g015290.3C* (*BrCER2*) required for C28 fatty acid elongation was strongly downregulated in HN-19G (**Table S9**).

## Discussion

### *BrCER2* is a gene controlling cuticular wax biosynthesis in Chinese cabbage

In Chinese cabbage, wax-less mutants showed a glossy green phenotype, distinctively different from the waxy glaucous plants. Previous studies showed that three genes have been identified for the glossy phenotype in Chinese cabbage. A single SNP in *Brcer1* (*Bra032670*) results in wax deficiency in Chinese cabbage (*B. rapa* L. ssp. *pekinensis*) (Liu et al., 2021). Subsequent research also delimited the locus related to the glossy phenotype to a 100.78-kb interval and showed that the *AtCER1* homolog *Bra032670* is the most likely candidate gene for *BrWAX2* (Yang et al., 2022b). The *BrWAX3* locus was fine-mapped to a 161.82-kb region on chromosome A09 of Chinese cabbage, with *Bra024749* (*BrCER60.A09*), encoding a β-ketoacyl-CoA synthase, identified as the candidate gene (Yang et al., 2022a). In a previous study, *BrCER2* was identified as the candidate gene for *BrWax1* (Zhang et al., 2013). An insertion at the transcription start site essentially silences *BrCER2* expression, thereby causing the mutant glossy phenotype of Chinese cabbage plants (Zhang et al., 2013). These candidate genes encode proteins with essential functions related to cuticular wax biosynthesis in Chinese cabbage. In the present study, a physical interval (130.1 kb) containing 20 genes was mapped and *BrCER2*, which is homologous to *AtCER2* (At4g24510), was identified as the candidate gene. Our SEM analysis generated evidence that *BrCER2* helps mediate cuticular wax biosynthesis in the cauline leaf. The results of the cuticular wax analysis indicated that a mutation to *BrCER2* affects more than C28 VLCFA biosynthesis and is responsible for the glossy phenotype of HN19-G. In *A. thaliana*, *Atcer2* mutant plants lack waxes longer than C28. Moreover, AtCER2 belongs to the BAHD acyltransferase family and is required for C28 elongation by interacting with fatty acid elongation machinery (Haslam et al., 2012). Co-expression of *AtCER2* with *AtCER6* in yeast results in the production of C30 fatty acids (Haslam et al., 2012). *CER2* of *Nelumbo nucifera* and *Oryza sativa* also showed similar functions in VLCFA biosynthesis (Wang et al., 2017; Yang et al., 2018), suggesting that the function of *CER2* in producing VLCFAs up to C30 is highly conserved across species.

### Retrotransposition of *BrLINE1-RUP* into *BrCER2* of HN19-G resulting in loss of *BrCER2* function

**Figure 8:**
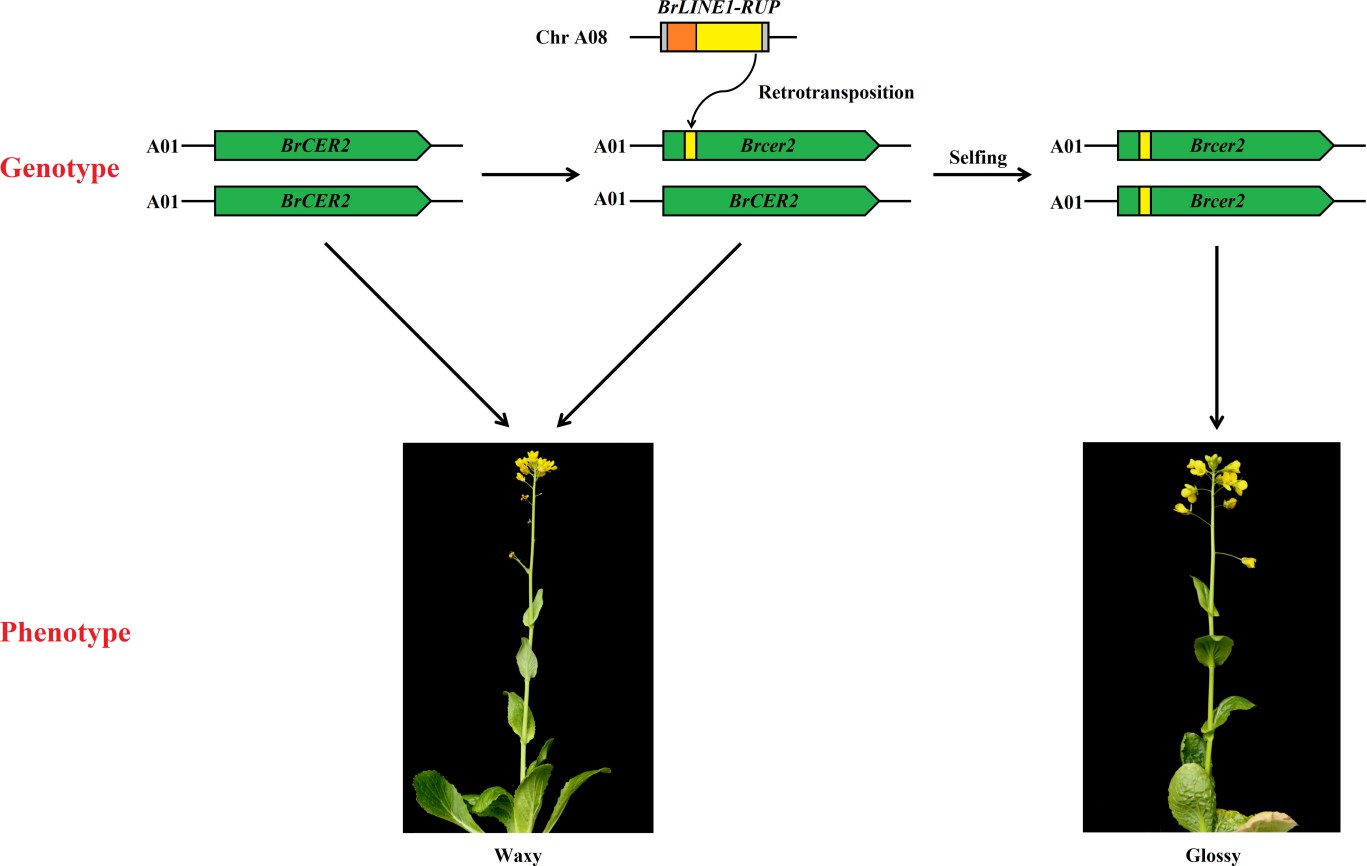
Schematic diagram of how the retrotransposition of *BrLINE1-RUP* altered the waxy phenotype of Chinese cabbage.

Transposable elements are potent broad-spectrum mutator elements that can increase genomic diversity (Gregory, 2011). Among *Brassica* species, the insertion of TEs is essential for phenotypic variations, adaptation, and domestication (Cai et al., 2021; Cai et al., 2022). A potential TE insertion was identified in exon 1 of *BrCER60.A09* in SD369, which lead to a premature stop codon, thus causing a loss of function of the BrCER60.A09 enzyme and a glossy phenotype in SD369 (Yang et al., 2022a). A copia-like retrotransposon-based marker (*BnSHP1.A9R2*) has been used for the marker-assisted breeding of oilseed rape with shatter-resistant pods (Liu et al., 2020). In yellowhorn (*Xanthoceras sorbifolium*), the *Xsag1-LINE1-1* fragment inserted in *XsAG1* is a *LINE-1* transposon; this fragment is responsible for the floral homeotic mutation in yellowhorn (Wang et al., 2022). In the current study, a 130-bp insertion in *Brcer2* of HN19-G was the result of the transposition of a sequence from *BrLINE1-RUP*, which is a LINE1 TE. More precisely, a retrotransposition event introduced a partial *BrLINE1-RUP* sequence (130 bp) into the first exon of *BrCER2* in HN19-G, thereby creating a premature termination codon in the *Brcer2* mRNA, ultimately leading to the formation of a truncated protein. A loss-of-function mutation to *BrCER2* causes the mutant Chinese cabbage plants to develop glossy cauline leaves rather than the normal waxy cauline leaves. Considered together, the study findings indicate the retrotransposition of *BrLINE1-RUP* into *BrCER2* modifies cuticular wax biosynthesis and affects the waxy phenotype (**Figure 8**). TE insertions play a crucial role in phenotypic variation and represent a major source of intraspecific variation.

### The transposition of *BrLINE1-RUP* into *Brcer2* of HN19-G probably involves an insertion-mediated deletion

The LINE-1 elements usually contain two ORFs, of which ORF1 encodes a nucleic acid-binding protein necessary for the retrotransposition of LINE1 elements. This protein functions as a nucleic acid chaperone that binds and preferentially mobilizes its own transcript (Callahan et al., 2012). In contrast, ORF2 encodes an endonuclease and a reverse transcriptase, the latter of which is essential for target-primed reverse transcription (Wells and Feschotte, 2020). In the present study, we determined that *BrLINE1-RUP* is missing ORF1. The insertion of *BrLINE1-RUP* into *BrCER2* of HN19-G suggests that ORF1 is not required for the transposition of *BrLINE1-RUP*. In accordance with this finding, ORF1 is reportedly dispensable or absent in some groups of non-LTR elements (Burke et al., 1987; Wells and Feschotte, 2020).

A 40-bp fragment was deleted from the first exon of *Brcer2.* The 130-bp insertion and the 40-bp deletion were localized to the same target site. Moreover, TSDs were not detected. The mechanism facilitating the transposition of *BrLINE1-RUP* is similar to that of L1IMDs, in which LINE-1 is inserted into a target site, while the target site sequence is removed. Earlier research confirmed L1IMDs occur in *Homo sapiens* and *Pan troglodytes* (Han et al., 2005). The insertion-mediated deletion-based transposition of *BrLINE1-RUP* provides evidence of L1IMDs in eukaryotes, including plants and animals.

**Figure 9:**
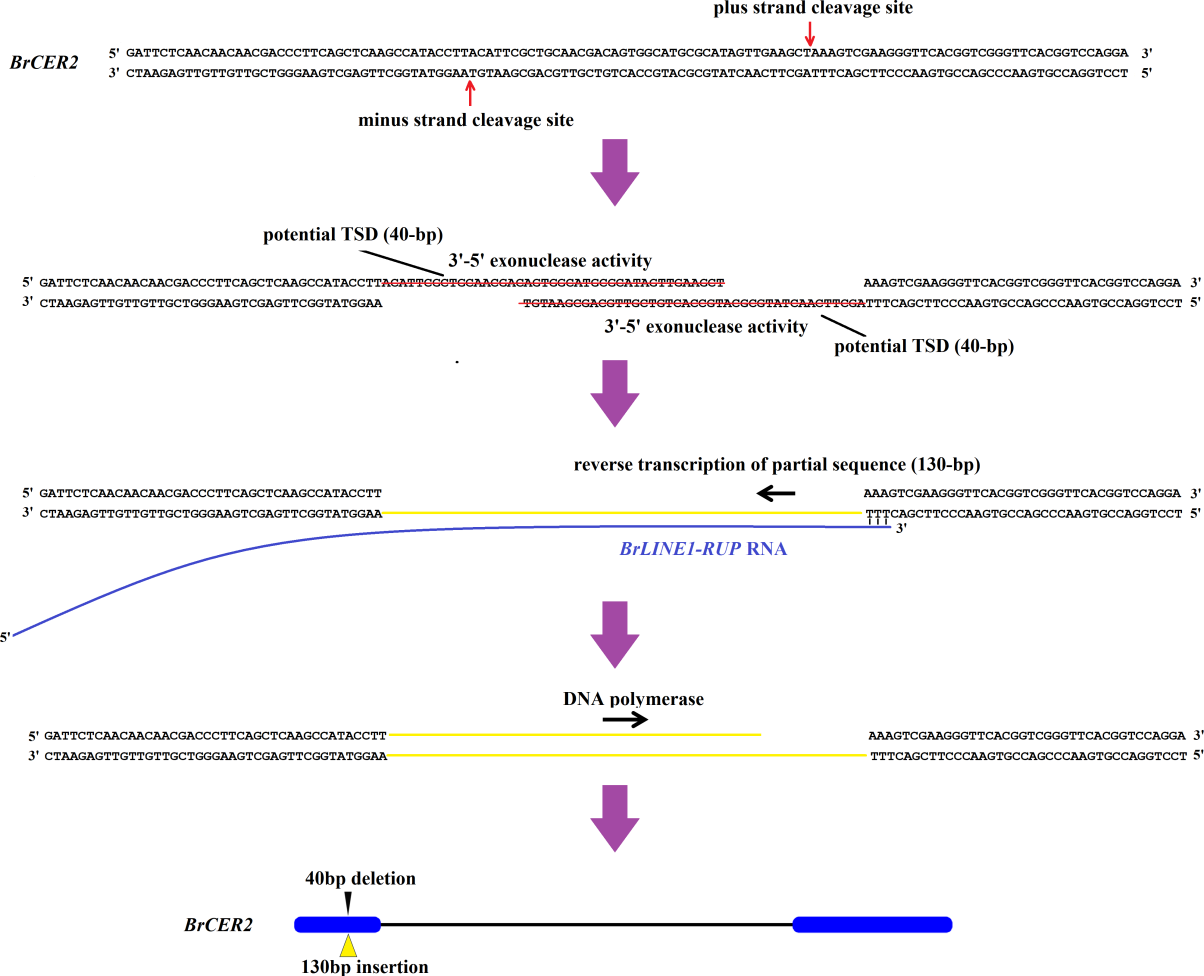
Putative model of the *BrLINE1-RUP* transposition into *Brcer2* of HN19-G via an insertion-mediated deletion mechanism.

The mechanism underlying L1IMDs was proposed to explain how a LINE-1 integration leads to target site deletions (Han et al., 2005). The *BrLINE1-RUP* sequence includes ORF2 (i.e., endonuclease and reverse transcriptase). The encoded endonuclease usually cleaves DNA at a 5′-TT/AAAA-3′ site, corresponding to genomic regions altered by LINE-1 integration (Richardson et al., 2015). In the present study, the 40-bp deletion in *Brcer2* coincided with the location of the integrated *BrLINE1-RUP.* The plus-strand cleavage site and the minus-strand cleavage site were respectively 5′-CT/AAAG-3′ and 5′-GT/AAGG-3′ (i.e., similar to 5′-TT/AAAA-3′). Moreover, 40-bp overhangs were produced and eliminated by the endonuclease (**Figure 9**). The poly(A) tail of the *BrLINE1-RUP* transcript can anneal to the cleavage site, thereby enabling the completion of target-primed reverse transcription. The *BrLINE1-RUP* sequence is 1,821 bp long, whereas the inserted fragment in *Brcer2* comprises 130 bp, suggesting that a partial *BrLINE1-RUP* RNA sequence was reverse transcribed during the retrotransposition of *BrLINE1-RUP* (**Figure 9**). A previous study showed that a hallmark feature of this process is the frequent premature termination of the reverse transcription step. The resulting 5′-truncation generally prevents the propagation of the newly inserted copy (Richardson et al., 2015; Wells and Feschotte, 2020).

## Data availability statement

The data presented in the study are deposited in the NCBI repository, accession number PRJNA967584 and PRJNA968036.

## Author contributions

PT performed most of the experiments and wrote the manuscript. BL initiated and directed the study. ZY performed genetic analysis. XD performed partial experiments. YaZ revised the manuscript. JL, YuZ, and QH collected partial data. All authors contributed to the article and approved the submitted version.
