# Supplementary figures and images for: A BrLINE1-RUP insertion in BrCER2 alters cuticular wax biosynthesis in Chinese cabbage (Brassica rapa L. ssp. pekinensis)
Source: Front Plant Sci. 2023 Jul 12;14:1212528. doi: 10.3389/fpls.2023.1212528 (PMC10368883; doi:10.3389/fpls.2023.1212528)

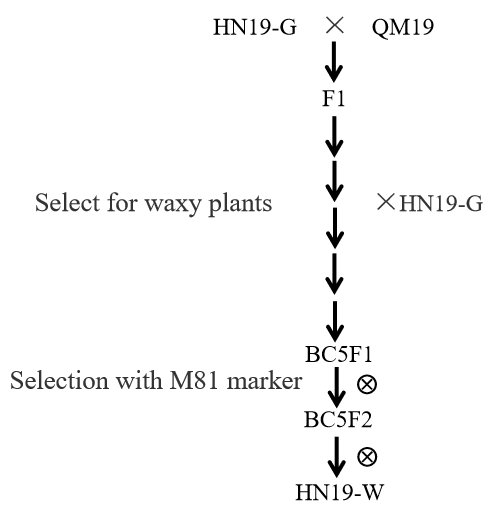


FIGURE S1 The detailed scheme for HN19-W NIL development

Supplement: Supplementary file 2 [file DataSheet_2.doc]

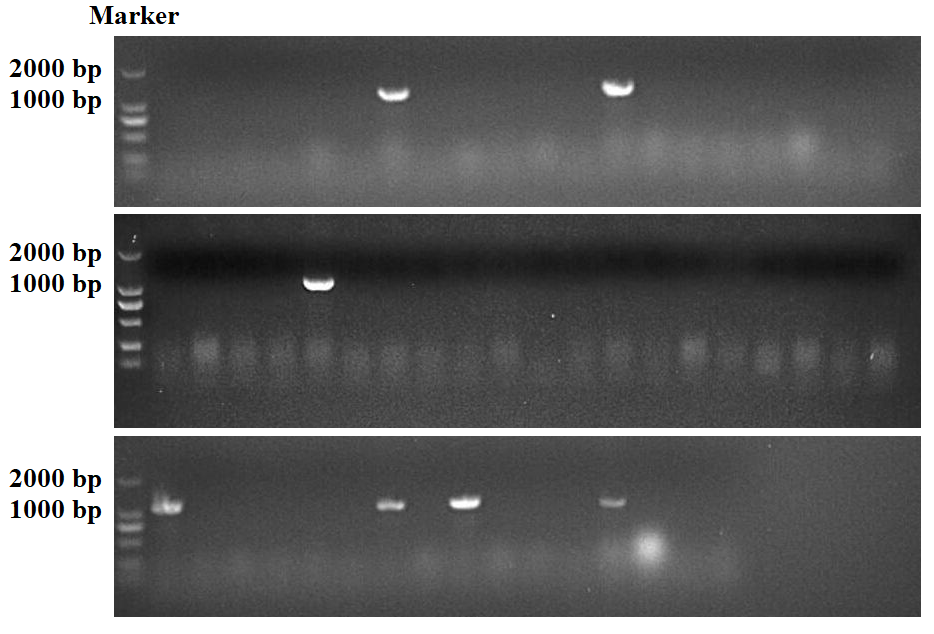


FIGURE S3 The PCR analysis of *BrLINE1-RUP* in 56 lines of *Brassica rapa* L. ssp. *Pekinensis.*

Supplement: Supplementary file 4 [file DataSheet_4.doc]
