# Supplementary material for: A BrLINE1-RUP insertion in BrCER2 alters cuticular wax biosynthesis in Chinese cabbage (Brassica rapa L. ssp. pekinensis)
Source: Front Plant Sci. 2023 Jul 12;14:1212528. doi: 10.3389/fpls.2023.1212528 (PMC10368883; doi:10.3389/fpls.2023.1212528)
Supplement: Supplementary file 3 [file DataSheet_3.doc]

>Chiifu *BrLINE1-RUP*

CATCACCATCATCAAGTCATGATTATTCCTCTGTCAAGGACTTTCCATCTTCAAAACGAATGAAGTCAGAATCTGACATTTTTTCTCCTTTTATTTGAAATACATAGAGCCAAGGCCTTAGCTAGCATTGTCTGTTAATATTCCACAACTAAGTTTTTTTGTTCATGCCTTTTTGTTTTGTGGTCAATACAAAGACTGTGTTTCTGGTTTTATTATCTTGTAAGGACTCAACTACTTCCTCCAGATTCTGGCGAGGTTTTGGTGTACTACACATTGACTGTGTATGCCATTTCTCTCATGTAGATGATCAGATACCTGAGAACTTTGTGATCTTGATCAGATACACATTGACTGTGTATGGCATTTTATACTATCTTAATACATCTCTCTCATGTAGTTGATCAGATACCTGAGAACTTAGTCTTGCTCTCCCAAAGAAAGTTACATGGAGATTCCTTCTAAGGCATAGTCTCGGAAGCTGCTTCGTTATGCTATTCAAGGTTCCACTTTCGGAATAACCTTCATCATCTCACAAACATACCACTGAGCTGAAGGGAGAATGATAAGATGGAGAGAAGAGAAAACTAGAGGAAGGAGCATTATAA*TATGGTTAACTAATGTTTTAAAGAAAAAAGTCTAAAAAAAAAATCAAAAAGCTCTCCAAAAAACGTTCTCTGATCGAAACTTATTTAAATCTCCGTCACCGACTCCGGTGGTTCGCCGCTACCGGAGTCGCCCTTCTCCTTTCCTATTTTTCTTTTTTGCTTCTCTTCCATCTCCTAGCTACCTTCCGATATGCTTGTTCTTGTGGGGTTCGCTCTTCCAAGCCCTAGATCCGGCCAGATCTGAGGTACGGCAGTTGCAGGTTCCTGGAGGCACGGCGAGGAGGTGAGGCAGGTGAAGGAGGTTGAGTCGGCTCTTAGGGTGGGGAAAGATTGGTTCGGCGGTTTTGTAGATCTAGATCTGCTTGTTCGGTCGTCTTTTCCAGAAGTGGCAGCACGTGAGCTAAGGAGCTTCCTCTTCTCGGATCTGTTCCTCGGGTTTGTAGTCGGAGTGGGGTGGTGGTTCGTTTGCGGTATCCTCGCCTTGGCGTCTTGGCTATGGAGCTTCTCTTGACTCCACCCAAGGTGGGTGTCGGGGTCTATTCTCCGGTGGCGCGTCTCAGTGGCTACCTTGGTTTCGTGTGTTGGCTGGTGTGTCTCTGTCGACGGCGCGTGGGGGTTCTCTGGCTGGTAAAGGCACGTGGAGTCGTCTATGGCTCTGGCGTCGGCTAGTCGAAGACCTTCCTCTGCCTTTGGATTTCTTCAGTTCTCGGTTTCTTCGTGTAGGAAGACCTGTTTGAGATTTTCGAGGCTTCTCTTCAGGTTTACTTTGGGTCTACTGGCCTCGGTTCCGTGCCTTTGACCTGGTACTTTGCAGGCACTGTCTCGGCGGTTGGAAGATGATTCGTTTCTCTGGTTCTCAGAGGCGTAACGTGCGGTTGAGTTTGGGTTTCTACGGTCATGAGGAGACCTTCACTTCACCGGTGGCGGCGTTGTTGAGTGTTGCCATCCCAGTGGCGATTTTGATCGTCAGGTTACTCTCTTTCTCTTCCATTGTAGGTCCCGGAGTTTTCTTATCAGTGGTGCGGCTGTGACTTCAACCCGCTTTTGTATAGCTTTTTGAGAGTAGTCGCTTCCTTGCTGACTTTGTGTCGGTCTGTGGAGATGTTGTGTAAGCCTGGTGGTCTCGTTACTATTGTTGCGGCATCTGCGGGTTTCTTGGTTGTGGTTTGCTGTTTAGTTTGTTGAGCTGTAGGAGGATGTAGTAAGGCTTCGGTGTGTGGAGATGCGGCTTTCCTTTATTTGTGGGCTCCTGCTATTAAGCTTCCTTGTTTCTGGTTCTGAGTGGAGATGCGACTCCTCTTTGTTTGTGAGTTCCTGCTATTAAGCTCGGGTGATCGTTTCTGGTTCCGAGTGGAGATGCGACTCCTTCTTCTTGTGAGTTCCTGCTATTAAGCTCAGGTGATCGTTATTCTGAAGTGGAGATGCGGCTCTTCTTTGCGAGTGAGCTCCTGCTATTAAGCCCCGTTAGCTTGTGTTGTCTTGGCGTTAGTCCCATCTTGGTCCTTCGTTTTTGCTGCCATTTGAATCAGCTTCCTATGTGTTCGCAATCGGTTAGCACCCTCCTAGTGTTCTTGTATGTGTTAGCCTTGGTCGAACCTCTTCTCCGGGAATACAGTCTTTATCGCTAATTCTCTAGGTGGATGCTTACAAGTTGAAGTTTGGAGAATTACTGATTATCGTTTGTTTCCATGTTTTTATCTTTTTTTGCCGTGGAATCAATTATCCTAGGTTCTACAATTTGTTAACAACAGTCTGTAAAACCTCTTGTTCATTTTTCGAAATGATATTTACATTTTTAGCAAAAAAAAATATGGTTAACTA*TCAGCAAATATAAATGCGAGAATATCTTGAGTAATCCATATTGATTATTATATGCTTAATTACGGACAAAAATATATGATATAAATGCGGATTTTTTAAAGTAATCCACATTAACTGAATTATAAGAGAGATGCAATATTTTAATTTTGGAGTAAATTATAAGAGCGTTGTAAAATTGTGAAATTTCATGCAAGAAGATTAACTAAAACAAACGAAATCGGTCGAAAACAAAACAGTTCTTATTTGTTTTCACTTTTATATTGAACCATAATATCAATTGTATATAATATAGCGAGAATATATTTTGGAGGATTAAAACAAAAGATTGGGAGGATATAAGATTTATGAGAATATATCGCATATGACAGAATTTGAAGAATTTTTAGATGAATAACTAAATTACAATGATACAAAAAGTAATATAACATACAAAGTAAGAGCATTTTCAAAATAGCAGTCATGTTTTAATAGTGTCGATGATCATAAAAGGAAAATTGACTTTCTAAAAGGTTTGAGAAGTTATGAGAAAAGCAAAGTAGTTGGAAAAAAAACTTATATAGTCAAAATTAAACGGAAATTATATCAACAAAAAGGAAAGTAACAACAAAACCTAAATAAATTAGGTTTCAAAGATCCTCCTAGACTAAGAAATTGGTATTGTACCCCTATAAATACCCCGCCCGTATTAAACTCTACAATTCATCACTGCAATCAAACAGAACAAAGCAAAAACCCTAAGAAACTATAGATATCGTTTTTCATTATGAGATCCAGAGGCGACTTGTATTTCAGGCATGAGATCGACCGTAACCCTTCAGCCTCGGATGCTGGCACATTCAAAGCATGCGCAACCATGGTCACATGGCACGAGGACATGGAAGATTCTGAAACAACATTCTCGTTTACGTTGTCGGCGGAGGACGTCATCGAACGGCAAGGACTTGCCAACAAAATACAAGAACTAGATGAGCTATTCATGGAAGCTGCTTTCCCTCAAGAAGATAATTTGCTTTTGTTGACCCAAGAAGCCTATCATTGTTTCATTGAAGTGATTTCCTCAAGTGATTATAGCAGAGATTGTGCTGTGTCTCTGTGGTTTACTTTTCGTGTTTGGCGTTCTCCTCCTCCTCTTCCTCCTTCAGTTGATGAATTTGAGGATGATGATGATGATGATGATGAGGCGACCAATAGTAATATTCCAATCAGGGCAGCAAGCAAGCTCGCCATCAAGTCCTTAACCAAGAAAATATACAACAAATGTGACTCTCTTGCCATTGACAGTTGCACTATTTGTTTGGAAGAGTTTAAGAGTGGAGTCAATGTTGTCGAGTTACCCTGTGGACATGAATTTGATGATGCATGTATCGGACACTGGTTCGAGACCAATCACATTTGTCCATTGTGTCGTTTCGAGTTGCCTCGTGAGCATCATTGA

>Z1 *BrLINE1-RUP*

CATCACCATCATCAAGTCATGATTATTCCTCTGTCAAGGACTTTCCATCTTCAAAACGAATGAAGTCAGAATCTGACATTTTTTCTCCTTTTATTTGAAATACATAGAGCGAAGGCCTTAGCTAGCATTGTCTGTTAATATTCCACAACTAAGTTTTTTTGTTCATGCCTTTTTGTTTTGTGGTCAATACAAAGACTGTGTTTCTGGTTTTATTATCTTGTAAGGACTCAACTACTTCCTCCAGATTCTGGCGAGGTTTTGGTGTACTACACATTGACTGTGTATGCCATTTCTCTCATGTAGATGATCAGATACCTGAGAACTTTGTGATCTTGATCAGATACACATTGACTGTGTATGGCATTTTATACTATCTTAATACATCTCTCTCATGTAGTTGATCAGATACCTGAGAACTTAGTCTTGCTCTCCCAAAGAAAGTTACATGGAGATTCCTTCTAAGGCATAGTCTCGGAAGCTGCTTCGTTATGCTATTCAAGGTTCCACTTTCGGAATAACCTTCATCATCTCACAAACATACCACTGAGCTGAAGGGAGAATGATAAGATGGAGAGAAGAGAAAACTAGAGGAAGGAGCATTATAA*TATGGTTAACTAATGTTTTAAAGAAAAAAGTCTAAAAAAAAAATCAAAAAGCTCTCCAAAAAACGTTCTCTGATCGAAACTTATTTAAATCTCCGTCACCGACTCCGGTGGTTCGCCGCTACCGGAGTCGCCCTTCTCCTTTCCTATTTTTCTTTTTTGCTTCTCTTCCATCTCCTAGCTACCTTCCGATATGCTTGTTCTTGTGGGGTTCGCTCTTCCAAGCCCTAGATCCGGCCAGATCTGAGGTACGGCAGTTGCAGGTTCCTGGAGGCACGGCGAGGAGGTGAGGCAGGTGAAGGAGGTTGAGTCGGCTCTTAGGGTGGGGAAAGATTGGTTCGGCGGTTTTGTAGATCTAGATCTGCTTGTTCGGTCGTCTTTTCCAGAAGTGGCAGCACGTGAGCTAAGGAGCTTCCTCTTCTCGGATCTGTTCCTCGGGTTTGTAGTCGGAGTGGGGTGGTGGTTCGTTTGCGGTATCCTCGCCTTGGCGTCTTGGCTATGGAGCTTCTCTTGACTCCACCCAAGGTGGGTGTCGGGGTCTATTCTCCGGTGGCGCGTCTCAGTGGCTACCTTGGTTTCGTGTGTTGGCTGGTGTGTCTCTGTCGACGGCGCGTGGGGGTTCTCTGGCTGGTAAAGGCACGTGGAGTCGTCTATGGCTCTAGCGTCGGCTAGTCGAAGACCTTCCTCTGCCTTTGGATTTCTTCAGTTCTCGGTTTCTTCGTGTAGGAAGACCTGATTGAGATTTTCGAGGCTTCTCTTCAGGTTTACTTTGGGTCTACTGGCCTCGGTTCCGTGCCTCTGACCTGGTACTTTGCAGGCACGGTCTCGGCGGTTGGAAGATGATTCGTTTCTCTGGTTCTCAGAGGCGTAACGTGCGGTTGAGTTTGGGTTTCTACGGTCATGAGGAGACCTTCACTTCACCGGTGGCGGCGTTGTTGAGTGTTGCCATCCCAGTGGCGATTTTGATCGTCAGGTTACTCTCTTTCTCTTCCATTGTAGGTCCCGGAGTTTTCTTATCAGTGGTGCGGCTGTGACTTCAACCCGCTTTTGTATAGCTTTTTGAGAGTAGTCGCTTCCTTGCTGACTTTGTGTCGGTCTGTGGAGATGTTGTGTAAGCCTGGTGGTCTCGTTACTATTGTTGCGGCATCTGCGGGTTTCTTGGTTGTGGTTTGCTGTTTAGTTTGTTGAGCTGTAGGAGGATGTAGTAAGGCTTCGGTGTGTGGAGATGCGGCTTTCCTTTATTTGTGGGCTCCTGCTATTAAGCTTCCTTGTTTCTGGTTCTGAGAGGAGATGCGACTCCTCTTTGTTTGTGAGTTCCTGCTATTAAGCTCGGGTGATCGTTTCTGGTTCCGAGTGGAGATGCGACTCCTTCTTCTTGTGAGTTCCTGCTATTAAGCTCAGGTGATCGTTATTCTGAAGTGGAGATGCGGCTCTTCTTTGCGAGTGAGCTCCTGCTATTAAGCCCCGTTAGCTTGTGTTGTCTTGGCGTTAGTCCCATCTTGGTCCTTCGTTTTTGCTGCCATTTGAATCAGCTTCCTATGTGTTCGCAATCGGTTAGCACCCTCCTAGTGTTCTTGTATGTGTTAGCCTTGGTCGAACCTCTTCTCCGGGAATACAGTCTTTATCGCTAATTCTCTAGGTGGATGCTTACAAGTTGAAGTTTGGAGAATTACTGATTATCGTTTGTTTCCATGTTTTTATCTTTTTTTGCCGTGGAATCAATTATCCTAGGTTCTACAATTTGTTAACAACAGTCTGTAAAACCTCTTGTTCATTTTTCGAAATGATATTTACATTTTTAGCAAAAAAAAATATGGTTAACTA*TCAGCAAATATAAATGCGAGAATATCTTGAGTAATCCATATTGATTATTATATGCTTAATTACGGACAAAAATATATGATATAAATGCGGATTTTTTAAAGTAATCCACATTAACTGAATTATAAGAGAGATGCAATATTTTAATTTTGGAGTAAATTATAAGAGCGTTGTAAAATTGTGAAATTTCATGCAAGAAGATTAACTAAAACAAACGAAATCGGTCGAAAACAAAACAGTTCTTATTTGTTTTCACTTTTATATTGAACCATAATATCAATTGTATATAATATAGCGAGAATATATTTTGGAGGATTAAAACAAAAGATTGGGAGGATATAAGATTTATGAGAATATATCGCATATGACAGAATTTGAAGAATTTTTAGATGAATAACTAAATTACAATGATACAAAAAGTAATATAACATACAAAGTAAGAGCATTTTCAAAATAGCAGTCATGTTTTAATAGTGTCGATGATCATAAAAGGAAAATTGACTTTCTAAAAGGTTTGAGAAGTTATGAGAAAAGCAAAGTAGTTGGAAAAAAAACTTATATAGTCAAAATTAAACGGAAATTATATCAACAAAAAGGAAAGTAACAACAAAACCTAAATAAATTAGGTTTCAAAGATCCTCCTAGACTAAGAAATTGGTATTGTACCCCTATAAATACCCCGCCCGTATTAAACTCTACAATTCATCACTGCAATCAAACAGAACAAAGCAAAAACCCTAAGAAACTATAGATATCGTTTTTCATTATGAGATCCAGAGGCGACTTGTATTTCAGGCATGAGATCGACCGTAACCCTTCAGCCTCGGATGCTGGCACATTCAAAGCATGCGCAACCATGGTCACATGGCACGAGGACATGGAAGATTCTGAAACAACATTCTCGTTTACGTTGTCGGCGGAGGACGTCATCGAACGGCAAGGACTTGCCAACAAAATACAAGAACTAGATGAGCTATTCATGGAAGCTGCTTTCCCTCAAGAAGATAATTTGCTTTTGTTGACCCAAGAAGCCTATCATTGTTTCATTGAAGTGATTTCCTCAAGTGATTATAGCAGAGATTGTGCTGTGTCTCTGTGGTTTACTTTTCGTGTTTGGCGTTCTCCTCCTCCTCTTCCTCCTTCAGTTGATGAATTTGAGGATGATGATGATGATGATGATGAGGCGACCAATAGTAATATTCCAATCAGGGCAGCAAGCAAGCTCGCCATCAAGTCCTTAACCAAGAAAATATACAACAAATGTGACTCTCTTGCCATTGACAGTTGCACTATTTGTTTGGAAGAGTTTAAGAGTGGAGTCAATGTTGTCGAGTTACCCTGTGGACATGAATTTGATGATGCATGTATCGGACACTGGTTCGAGACCAATCACATTTGTCCATTGTGTCGTTTCGAGTTGCCTCGTGAGCATCATTGA

>TBA *BrLINE1-RUP*

CCTCATCATCATCAAGTCATGATGATTCCTCTGTCAAGGACTTTCCATCTTCAAAACGAATGAAGTCAGAATCTGACATTTTTTCTCCTTTCATTTGAAATACTTAGAGCCAAGACCTTAGCTAGCATTGCCTGTTAATATTCCACAACTAAGTTTTTCTGTTCATGCCTTTTTGTTTTGTGGTCAATACAAAGACTGTGTTTCTGGTTTTGTTATCTTGTAAGAACTCAACTACTTCCTCCAGATTCTGGCGAGGGTTTGGTGTACTACACATTGACTGTGTATGCCATTTCCTCATGTAGTTGATCAGATACCTGAGAACTTAGTGATCTTGATCAGATACACATTGACTGTGTATGGCATTTTATACTATCTTAATACATCTCTCTCATGTAGTTGATCAGATACCTGAGAACTTAGTCTTGCTCTCCCAAAGAAAGTTACATGGAGATTCCTTTTAAGGCATAGTCTCGGAAGCTGCTTCGTTATGCTATTCAAGGTTCCACTTTCGGAATAACCTTCATCATCACAAACATACCACTGAGCTGAAGGGAGAATGATAAGATGGAGAGAAGAGAAAACTAGAGGAAGGACTAAGGAGCATTATATGGTTAACTATCAGCAAAAGTATACAAATATAAATGCGAGAATATCTTGAGTAATCCATATTGATTATATTATATGTTTAATTATGGACAAAAATATTTGATATAAATGCGGATTTTTGAAGTAATCCACATTAACTGAATTATAAGAGCGATGCAATATTTTAATTTTGGACTAAATTATAAGAGCGTTGTAAAATTGTGAAATTTCATGCAAGAAGATTAACTAAAACAAACGAAATCGGTGGAAAACAAAACAGTTCTTATTTGTTTTCACTTTTATATTGAACCATAATATCAATTGTATACTATAATATAGCGAGAATATATTTTGGAGGAATTAAAACAAAAGATTGGGAGGATATAAGATTTATGAGAATATCTCGCATATGACAGAATTTGAAGAATTTTTAGATGAATAACTAAATTACAATGATACAAAAAGTAATATAACATACAAAGCAAGAGCATTTTCAAAATAGCAATCATGTTTTAATAGTGTCGATGATCATAAAAGGAAAATTGACTTTCTAAAAGGTTTGAGAAGTTATTAGAAAAGCAAAGTAGTTGAAAAAAAAACTTATATAGTCAAAATTAAACGGAAATTATATCAACAAAAGGGAAAGTAACAACAAAACCTAAATAAATTAGTATGATCTTGTTTCTAAAAAAACCCTAAGAAACTATAGATATCGTTTTTCATTATGAGATCTAGAGGCGACTTGTATTTCAGGCATGAGATCGACCGTAACCCTTAAGCCTCGGATGCTGGCACAATCAAAGCATGCGCAACCATGGTCACATGGCAGGAGGACATGGCATATTTTGAAACAACATTCTCGTTTACGTTGTCGGCGGAGGACGTCATCGAACGCCAAAGACTTGCCAACAAAATACAAGAACTAGATGAGTTATTCATGGAATCTGCTTTCCCTCGAGAAGATAATTTGCTTTTGTTGACCCAAGAAGCCTATCATTGTTTCATTAAATTGATTTCCTCACGTGATTATAGTAGAGATTGTGTTATGTCTCTGTGGTTTACTTTTCGTGTTTGGCGTACTCCTCTTCCTCCTTCAATTGATGAATGTGAGGATGATGATGATGATGAGGCGACCAATAGTAATATTCCAATCAGGACAGCAAGCAAGCTCGCGGTCAAGTCCTTAACCAAGAAAATATACAACAAAGGTGACTCTCTCGCCATTGACAGTTGCACTATTTATACCCGGTTGGAAGGGTTAATCACGACCTTTATATCCGGTCGGAAGGGTTAA

>BRO *BrLINE1-RUP*

CATCACCATCATCAAGTCATGATTATTCCTCTGTCAAGGACTTTCCATCTTCAAAACGAATGAAGTCAGAATCTGACATTTTTTCTCCTTTTATTTGAAATACATAGAGCCAAGGCCTTAGCTAGCATTGTCTGTTAATATTCCACAACTAAGTTTTTTTGTTCATGCCTTTTTGTTTTGTGGTCAATACAAAGACTGTGTTTCTGGTTTTATTATCTTGTAAGGACTCAACTACTTCCTCCAGATTCTGGCGAGGTTTTGGTGTACTACACATTGACTGTGTATGCCATTTCTCTCATGTAGATGATCAGATACCTGAGAACTTTGTGATCTTGATCAGATACACATTGACTGTGTATGGCATTTTATACTATCTTAATACATCTCTCTCATGTAGTTGATCAGATACCTGAGAACTTAGTCTTGCTCTCCCAAAGAAAGTTACATGGAGATTCCTTCTAAGGCATAGTCTCGGAAGCTGCTTCGTTATGCTATTCAAGGTTCCACTTTCGGAATAACCTTCATCATCTCACAAACATACCACTGAGCTGAAGGGAGAATGATAAGATGGAGAGAAGAGAAAACTAGAGGAAGGAGCATTATAA*TATGGTTAACTAATGTTTTAAAGAAAAAAGTCTAAAAAAAAAATCAAAAAGCTCTCCAAAAAACGTTCTCTGATCGAAACTTATTTAAATCTCCGTCACCGACTCCGGTGGTTCGCCGCTACCGGAGTCGCCCTTCTCCTTTCCTATTTTTCTTTTTTGCTTCTCTTCCATCTCCTAGCTACCTTCCGATATGCTTGTTCTTGTGGGGTTCGCTCTTCCAAGCCCTAGATCCGGCCAGATCTGAGGTACGGCAGTTGCAGGTTCCTGGAGGCACGGCGAGGAGGTGAGGCAGGTGAAGGAGGTTGAGTCGGCTCTTAGGGTGGGGAAAGATTGGTTCGGCGGTTTTGTAGATCTAGATCTGCTTGTTCGGTCGTCTTTTCCAGAAGTGGCAGCACGTGAGCTAAGGAGCTTCCTCTTCTCGGATCTGTTCCTCGGGTTTGTAGTCGGAGTGGGGTGGTGGTTCGTTTGCGGTATCCTCGCCTTGGCGTCTTGGCTATGGAGCTTCTCTTGACTCCACCCAAGGTGGGTGTCGGGGTCTATTCTCCGGTGGCGCGTCTCAGTGGCTACCTTGGTTTCGTGTGTTGGCTGGTGTGTCTCTGTCGACGGCGCGTGGGGGGTTCTCTGGCTGGTAAAGGCACGTGGAGTCGTCTATGGCTCTAGCGTCGGCTAGTCGAAGACCTTCCTCTGCCTTTGGATTTCTTCAGTTCTCGGTTTCTTCGTGTAGGAAGACCTGATTGAGATTTTCGAGGCTTCTCTTCAGGTTTACTTTGGGTCTACTGGCCTCGGTTCCGTGCCTCTGACCTGGTACTTTGCAGGCACGGTCTCGGCGGTTGGAAGATGATTCGTTTCTCTGGTTCTCAGAGGCGTAACGTGCGGTTGAGTTTGGGTTTCTACGGTCATGAGGAGACCTTCACTTCACCGGTGGCGGCGTTGTTGAGTGTTGCCATCCCAGTGGCGATTTTGATCGTCAGGTTACTCTCTTTCTCTTCCATTGTAGGTCCCGGAGTTTTCTTATCAGTGGTGCGGCTGTGACTTCAACCCGCTTTTGTATAGCTTTTTGAGAGTAGTCGCTTCCTTGCTGACTTTGTGTCGGTCTGTGGAGATGTTGTGTAAGCCTGGTGGTCTCGTTACTATTGTTGCGGCATCTGCGGGTTTCTTGGTTGTGGTTTGCTGTTTAGTTTGTTGAGCTGTAGGAGGATGTAGTAAGGCTTCGGTGTGTGGAGATGCGGCTTTCCTTTATTTGTGGGCTCCTGCTATTAAGCTTCCTTGTTTCTGGTTCTGAGAGGAGATGCGACTCCTCTTTGTTTGTGAGTTCCTGCTATTAAGCTCGGGTGATCGTTTCTGGTTCCGAGTGGAGATGCGACTCCTTCTTCTTGTGAGTTCCTGCTATTAAGCTCAGGTGATCGTTATTCTGAAGTGGAGATGCGGCTCTTCTTTGCGAGTGAGCTCCTGCTATTAAGCCCCGTTAGCTTGTGTTGTCTTGGCGTTAGTCCCATCTTGGTCCTTCGTTTTTGCTGCCATTTGAATCAGCTTCCTATGTGTTCGCAATCGGTTAGCACCCTCCTAGTGTTCTTGTATGTGTTAGCCTTGGTCGAACCTCTTCTCCGGGAATACAGTCTTTATCGCTAATTCTCTAGGTGGATGCTTACAAGTTGAAGTTTGGAGAATTACTGATTATCGTTTGTTTCCATGTTTTTATCTTTTTTTGCCGTGGAATCAATTATCCTAGGTTCTACAATTTGTTAACAACAGTCTGTAAAACCTCTTGTTCATTTTTCGAAATGATATTTACATTTTTAGCAAAAAAAAATATGGTTAACTA*TCAGCAAATATAAATGCGAGAATATCTTGAGTAATCCATATTGATTATTATATGCTTAATTACGGACAAAAATATATGATATAAATGCGGATTTTTTAAAGTAATCCACATTAACTGAATTATAAGAGAGATGCAATATTTTAATTTTGGAGTAAATTATAAGAGCGTTGTAAAATTGTGAAATTTCATGCAAGAAGATTAACTAAAACAAACGAAATCGGTCGAAAACAAAACAGTTCTTATTTGTTTTCACTTTTATATTGAACCATAATATCAATTGTATATAATATAGCGAGAATATATTTTGGAGGATTAAAACAAAAGATTGGGAGGATATAAGATTTATGAGAATATATCGCATATGACAGAATTTGAAGAATTTTTAGATGAATAACTAAATTACAATGATACAAAAAGTAATATAACATACAAAGTAAGAGCATTTTCAAAATAGCAGTCATGTTTTAATAGTGTCGATGATCATAAAAGGAAAATTGACTTTCTAAAAGGTTTGAGAAGTTATGAGAAAAGCAAAGTAGTTGGAAAAAAAACTTATATAGTCAAAATTAAACGGAAATTATATCAACAAAAAGGAAAGTAACAACAAAACCTAAATAAATTAGGTTTCAAAGATCCTCCTAGACTAAGAAATTGGTATTGTACCCCTATAAATACCCCGCCCGTATTAAACTCTACAATTCATCACTGCAATCAAACAGAACAAAGCAAAAACCCTAAGAAACTATAGATATCGTTTTTCATTATGAGATCCAGAGGCGACTTGTATTTCAGGCATGAGATCGACCGTAACCCTTCAGCCTCGGATGCTGGCACATTCAAAGCATGCGCAACCATGGTCACATGGCACGAGGACATGGAAGATTCTGAAACAACATTCTCGTTTACGTTGTCGGCGGAGGACGTCATCGAACGGCAAGGACTTGCCAACAAAATACAAGAACTAGATGAGCTATTCATGGAAGCTGCTTTCCCTCAAGAAGATAATTTGCTTTTGTTGACCCAAGAAGCCTATCATTGTTTCATTGAAGTGATTTCCTCAAGTGATTATAGCAGAGATTGTGCTGTGTCTCTGTGGTTTACTTTTCGTGTTTGGCGTTCTCCTCCTCCTCTTCCTCCTTCAGTTGATGAATTTGAGGATGATGATGATGATGATGATGAGGCGACCAATAGTAATATTCCAATCAGGGCAGCAAGCAAGCTCGCCATCAAGTCCTTAACCAAGAAAATATACAACAAATGTGACTCTCTTGCCATTGACAGTTGCACTATTTGTTTGGAAGAGTTTAAGAGTGGAGTCAATGTTGTCGAGTTACCCTGTGGACATGAATTTGATGATGCATGTATCGGACACTGGTTCGAGACCAATCACATTTGTCCATTGTGTCGTTTCGAGTTGCCTCGTGAGCATCATTGA

>CCA *BrLINE1-RUP*

CATCACCATCATCAAGTCATGATTATTCCTCTGTCAAGGACTTTCCATCTTCAAAACGAATGAAGTCAGAATCTGACATTTTTTCTCCTTTTATTTGAAATACATAGAGCCAAGGCCTTAGCTAGCATTGTCTGTTAATATTCCACAACTAAGTTTTTTTGTTCATGCCTTTTTGTTTTGTGGTCAATACAAAGACTGTGTTTCTGGTTTTATTATCTTGTAAGGACTCAACTACTTCCTCCAGATTCTGGCGAGGTTTTGGTGTACTACACATTGACTGTGTATGCCATTTCTCTCATGTAGATGATCAGATACCTGAGAACTTTGTGATCTTGATCAGATACACATTGACTGTGTATGGCATTTTATACTATCTTAATACATCTCTCTCATGTAGTTGATCAGATACCTGAGAACTTAGTCTTGCTCTCCCAAAGAAAGTTACATGGAGATTCCTTCTAAGGCATAGTCTCGGAAGCTGCTTCGTTATGCTATTCAAGGTTCCACTTTCGGAATAACCTTCATCATCTCACAAACATACCACTGAGCTGAAGGGAGAATGATAAGATGGAGAGAAGAGAAAACTAGAGGAAGGAGCATTATAA*TATGGTTAACTAATGTTTTAAAGAAAAAAGTCTAAAAAAAAAATCAAAAAGCTCTCCAAAAAACGTTCTCTGATCGAAACTTATTTAAATCTCCGTCACCGACTCCGGTGGTTCGCCGCTACCGGAGTCGCCCTTCTCCTTTCCTATTTTTCTTTTTTGCTTCTCTTCCATCTCCTAGCTACCTTCCGATATGCTTGTTCTTGTGGGGTTCGCTCTTCCAAGCCCTAGATCCGGCCAGATCTGAGGTACGGCAGTTGCAGGTTCCTGGAGGCACGGCGAGGAGGTGAGGCAGGTGAAGGAGGTTGAGTCGGCTCTTAGGGTGGGGAAAGATTGGTTCGGCGGTTTTGTAGATCTAGATCTGCTTGTTCGGTCGTCTTTTCCAGAAGTGGCAGCACGTGAGCTAAGGAGCTTCCTCTTCTCGGATCTGTTCCTCGGGTTTGTAGTCGGAGTGGGGTGGTGGTTCGTTTGCGGTATCCTCGCCTTGGCGTCTTGGCTATGGAGCTTCTCTTGACTCCACCCAAGGTGGGTGTCGGGGTCTATTCTCCGGTGGCGCGTCTCAGTGGCTACCTTGGTTTCGTGTGTTGGCTGGTGTGTCTCTGTCGACGGCGCGTGGGGGGTTCTCTGGCTGGTAAAGGCACGTGGAGTCGTCTATGGCTCTAGCGTCGGCTAGTCGAAGACCTTCCTCTGCCTTTGGATTTCTTCAGTTCTCGGTTTCTTCGTGTAGGAAGACCTGATTGAGATTTTCGAGGCTTCTCTTCAGGTTTACTTTGGGTCTACTGGCCTCGGTTCCGTGCCTCTGACCTGGTACTTTGCAGGCACGGTCTCGGCGGTTGGAAGATGATTCGTTTCTCTGGTTCTCAGAGGCGTAACGTGCGGTTGAGTTTGGGTTTCTACGGTCATGAGGAGACCTTCACTTCACCGGTGGCGGCGTTGTTGAGTGTTGCCATCCCAGTGGCGATTTTGATCGTCAGGTTACTCTCTTTCTCTTCCATTGTAGGTCCCGGAGTTTTCTTATCAGTGGTGCGGCTGTGACTTCAACCCGCTTTTGTATAGCTTTTTGAGAGTAGTCGCTTCCTTGCTGACTTTGTGTCGGTCTGTGGAGATGTTGTGTAAGCCTGGTGGTCTCGTTACTATTGTTGCGGCATCTGCGGGTTTCTTGGTTGTGGTTTGCTGTTTAGTTTGTTGAGCTGTAGGAGGATGTAGTAAGGCTTCGGTGTGTGGAGATGCGGCTTTCCTTTATTTGTGGGCTCCTGCTATTAAGCTTCCTTGTTTCTGGTTCTGAGAGGAGATGCGACTCCTCTTTGTTTGTGAGTTCCTGCTATTAAGCTCGGGTGATCGTTTCTGGTTCCGAGTGGAGATGCGACTCCTTCTTCTTGTGAGTTCCTGCTATTAAGCTCAGGTGATCGTTATTCTGAAGTGGAGATGCGGCTCTTCTTTGCGAGTGAGCTCCTGCTATTAAGCCCCGTTAGCTTGTGTTGTCTTGGCGTTAGTCCCATCTTGGTCCTTCGTTTTTGCTGCCATTTGAATCAGCTTCCTATGTGTTCGCAATCGGTTAGCACCCTCCTAGTGTTCTTGTATGTGTTAGCCTTGGTCGAACCTCTTCTCCGGGAATACAGTCTTTATCGCTAATTCTCTAGGTGGATGCTTACAAGTTGAAGTTTGGAGAATTACTGATTATCGTTTGTTTCCATGTTTTTATCTTTTTTTGCCGTGGAATCAATTATCCTAGGTTCTACAATTTGTTAACAACAGTCTGTAAAACCTCTTGTTCATTTTTCGAAATGATATTTACATTTTTAGCAAAAAAAAATATGGTTAACTA*TCAGCAAATATAAATGCGAGAATATCTTGAGTAATCCATATTGATTATTATATGCTTAATTACGGACAAAAATATATGATATAAATGCGGATTTTTTAAAGTAATCCACATTAACTGAATTATAAGAGAGATGCAATATTTTAATTTTGGAGTAAATTATAAGAGCGTTGTAAAATTGTGAAATTTCATGCAAGAAGATTAACTAAAACAAACGAAATCGGTCGAAAACAAAACAGTTCTTATTTGTTTTCACTTTTATATTGAACCATAATATCAATTGTATATAATATAGCGAGAATATATTTTGGAGGATTAAAACAAAAGATTGGGAGGATATAAGATTTATGAGAATATATCGCATATGACAGAATTTGAAGAATTTTTAGATGAATAACTAAATTACAATGATACAAAAAGTAATATAACATACAAAGTAAGAGCATTTTCAAAATAGCAGTCATGTTTTAATAGTGTCGATGATCATAAAAGGAAAATTGACTTTCTAAAAGGTTTGAGAAGTTATGAGAAAAGCAAAGTAGTTGGAAAAAAAACTTATATAGTCAAAATTAAACGGAAATTATATCAACAAAAAGGAAAGTAACAACAAAACCTAAATAAATTAGGTTTCAAAGATCCTCCTAGACTAAGAAATTGGTATTGTACCCCTATAAATACCCCGCCCGTATTAAACTCTACAATTCATCACTGCAATCAAACAGAACAAAGCAAAAACCCTAAGAAACTATAGATATCGTTTTTCATTATGAGATCCAGAGGCGACTTGTATTTCAGGCATGAGATCGACCGTAACCCTTCAGCCTCGGATGCTGGCACATTCAAAGCATGCGCAACCATGGTCACATGGCACGAGGACATGGAAGATTCTGAAACAACATTCTCGTTTACGTTGTCGGCGGAGGACGTCATCGAACGGCAAGGACTTGCCAACAAAATACAAGAACTAGATGAGCTATTCATGGAAGCTGCTTTCCCTCAAGAAGATAATTTGCTTTTGTTGACCCAAGAAGCCTATCATTGTTTCATTGAAGTGATTTCCTCAAGTGATTATAGCAGAGATTGTGCTGTGTCTCTGTGGTTTACTTTTCGTGTTTGGCGTTCTCCTCCTCCTCTTCCTCCTTCAGTTGATGAATTTGAGGATGATGATGATGATGATGATGAGGCGACCAATAGTAATATTCCAATCAGGGCAGCAAGCAAGCTCGCCATCAAGTCCTTAACCAAGAAAATATACAACAAATGTGACTCTCTTGCCATTGACAGTTGCACTATTTGTTTGGAAGAGTTTAAGAGTGGAGTCAATGTTGTCGAGTTACCCTGTGGACATGAATTTGATGATGCATGTATCGGACACTGGTTCGAGACCAATCACATTTGTCCATTGTGTCGTTTCGAGTTGCCTCGTGAGCATCATTGA

>CCB *BrLINE1-RUP*

CCTCATCATCATCATCAAGTCATGATGATTCCTCTGTCAAGGACTTTCCATCTTCAAAACGAATGAAGTCAGAATCTGACATTTTTTCTCCGTTTATTTGAAATACATAGAGCCACGACCTTAGCTAGCATTGTCTGTTAATATTCCACATCTAAGTTTTTTTGTTCATGCCTTTTTGTTTTGTGTTCAATACAAAGACTGTGTTTCTGGTTTTGTTATCTTGTAAGAAGAACTCAACTACTTCCTCCAGATTCTGGCGAGGGTTTGGTGTACTACACATTGACTGTGTATGCCATTTCTCTCATGTAGTTGATCAGATACCTGAGAACTTAGTGATCTTGATCAGATACACATTGACTGTGTATGGCATTTTATACTATCTTAATACATCTCTCTCATGTAGTTGATCAGATACCTGAGAACTTAGTCTTGCTCTCCCAAAGAAAGTTACATGGAGATTCCTTTTAAGGCATAGTCTCGGAAGCTGCTTCGTTATGCTATTCAAGGTTCCACTTTCGGAATAACCTTCATCATCACAAACATACCACTGAGCTGAAGGGAGAATAAAAAGATGGAGAAAAGAGAAAACTAGAGGAAGGAGCATTATATGGTTAACTATCAGCAAAAGTATACAAATATAAATGCGAGAATATCTTGAGTAATCCATATTGATTATATTATATGCTTAATTATGGACAAAAATATATGATATAAATGCGGATTTTTTTAAGTAATCCACATTAACTGAATTATAAGAGCGATGCAATATTTTAATTTTGGACTAAATTATAAGAGCGTTGTAAAATTGTAAAATTTCATGCAAGAAGATTAACTAAAACAAACGAAATCGGTGGAAAACAAAATAGTTCTTATTTGTTTTCACTTTTATATTGAACCATAATATCAATTGTATATAATATAGAGAGAATATATTTTGGAGGAATTAAAACAAAAGATTGGGAGGATATAAGATTTATGAGAATATCTCGCATATGACAGATTTTGAAGAGTTTTTAGATAAATAACTAAATTACAATGATACAAAAAGTAATATAACATACAAAGTAAGAGCATTTTCAAAATAGCAATCATGTTTTAATAGTGTCGATGATCATAAAAGGAAAATTGACTTTCTAAAAGGTTTGAGAAGTTATGAGAAAAGCAAAGTAATTGGGAAAAAACCTTATATAGTCAAAATTAAACGGAAATTATATCAACAAAAAGGAAAGTAACAACAAAACCTAAATAAATTAGTATGATCTTGTTTCTAAAAAAAACCCTAAGAAACTATAGATATCGTTTTTCATTATGAGATCTAGAGGCGACTTGTATTTCAGGCATGAGATCGACCATAACCCTTAAGCCTCGGATGCTGGCACAATCAAAGCATGCGCAACCATGGTCACATGGCACGAGGACATGGAAGATTCTGAAACAACATTCTCGTTTACGTTGTCGGCGGAGGACGTCATCGAACGCCAAGGACTTGCCAACAAAATACAAGAACTAGATGAGTTATTCATGGAATCTGCTTTCCCTCGAGAAGATAATTTGCTTTTGTTGACCCAAGAAGCCTATCATTGTTTCATTAAATTGATTTCCTCACGTGATTATAGCAGAGATTGTGCTATGTCTCTGTGGTTTACTTTTCGTGTTTGGCGTACTCTTCTTCCTCCTTCAGTTGATGAATTTGAGGATGATGATGATGATGAGGCGACCAATAGTAATATTCCAATCAGGGCAGCAAGCTCGCGGTCAAGTCCTTAACCAAGAAAATATACAACAAAGGTGACTCTCTCGCCATTGACAGTTGCACTATTTATACCCGGTTGAAAGGGTTAATCACGACCTTTATATCCGGTCGGAAGGGTTAA

>CXA *BrLINE1-RUP*

CATCATCATCATCAAGTCATGATGATTCCTCTGTCAAGGACTTTCCATCTTCAAAACGAATGAAGTCAGAATCTGACATTTTTTCTCCGTTTATTTGAAATACATAGAGCCACGACCTTAGCTAGCATTGTCTGTTAATATTCCACATCTAAGTTTTTTTGTTCATGCCTTTTTGTTTTGTGTTCAATACAAAGACTGTGTTTCTGGTTTTGTTATCTTGTAAGAAGAACTCAACTACTTCCTCCAGATTCTGGCGAGGGTTTGGTGTACTACACATTGACTGTGTATGCCATTTCTCTCATGTAGTTGATCAGATACCTGAGAACTTAGTGATCTTGATCAGATACACATTGACTGTGTATGGCATTTTATACTATCTTAATACATCTCTCTCATGTAGTTGATCAGATACCTGAGAACTTAGTCTTGCTCTCCCAAAGAAAGTTACATGGAGATTCCTTTTAAGGCATAGTCTCGGAAGCTGCTTCGTTATGCTATTCAAGGTTCCACTTTCGGAATAACCTTCATCATCACAAACATACCACTGAGCTGAAGGGAGAATAAAAAGATGGAGAAAAGAGAAAACTAGAGGAAGGAGCATTATATGGTTAACTATCAGCAAAAGTATACAAATATAAATGCGAGAATATCTTGAGTAATCCATATTGATTATATTATATGCTTAATTATGGATAAAAATATATGATATAAATGCGGATTTTTTTAAGTAATCCACATTAACTGAATTATAAGAGCGATGCAATATTTTAATTTTGGACTAAATTATAAGAGCGTTGTAAAATTGTAAAATTTCATGCAAGAAGATTAACTAAAACAAACGAAATCGGTGGAAAACAAAATAGTTCTTATTTGTTTTCACTTTTATATTGAACCATAATATCAATTGTATATAATATAGAGAGAATATATTTTGGAGGAATTAAAACAAAAGATTGGGAGGATATAAGATTTATGAGAATATCTCGCATATGACAGATTTTGAAGAGTTTTTAGATAAATAACTAAATTACAATGATACAAAAAGTAATATAACATACAAAGTAAGAGCATTTTCAAAATAGCAATCATGTTTTAATAGTGTCGATGATCATAAAAGGAAAATTGACTTTCTAAAAGGTTTGAGAAGTTATGAGAAAAGCAAAGTAATTGGGAAAAAACCTTATATAGTCAAAATTAAACGGAAATTATATCAACAAAAAGGAAAGTAACAACAAAACCTAAATAAATTAGTATGATCTTGTTTCTAAAAAAAACCCTAAGAAACTATAGATATCGTTTTTCATTATGAGATCTAGAGGCGACTTGTATTTCAGGCATGAGATCGACCATAACCCTTAAGCCTCGGATGCTGGCACAATCAAAGCATGCGCAACCATGGTCACATGGCACGAGGACATGGAAGATTCTGAAACAACATTCTCGTTTACGTTGTCGGCGGAGGACATCATCGAACGCCAAGGACTTGCCAACAAAATACAAGAACTAGATGAGTTATTCATGGAATCTGCTTTCCCTCGAGAAGATAATTTGCTTTTGTTGACCCAAGAAGCCTATCATTGTTTCATTAAATTGATTTCCTCACGTGATTATAGCAGAGATTGTGCTATGTCTCTGTGGTTTACTTTTCGTGTTTGGCGTACTCTTCTTCCTCCTTCAGTTGATGAATTTGAGGATGATGATGATGATGAGGCGACCAATAGTAATATTCCAATCAGGGCAGCAAGCTCGCGGTCAAGTCCTTAACCAAGAAAATATACAACAAAGGTGACTCTCTCGCCATTGACAGTTGCACTATTTATACCCGGTTGAAAGGGTTAATCACGACCTTTATATCCGGTCGGAAGGGTTAA

>CXB *BrLINE1-RUP*

CATCACCATCATCAAGTCATGATTATTCCTCTGTCAAGGACTTTCCATCTTCAAAACGAATGAAGTCAGAATCTGACATTTTTTCTCCTTTTATTTGAAATACATAGAGCCAAGGCCTTAGCTAGCATTGTCTGTTAATATTCCACAACTAAGTTTTTTTGTTCATGCCTTTTTGTTTTGTGGTCAATACAAAGACTGTGTTTCTGGTTTTATTATCTTGTAAGGACTCAACTACTTCCTCCAGATTCTGGCGAGGTTTTGGTGTACTACACATTGACTGTGTATGCCATTTCTCTCATGTAGATGATCAGATACCTGAGAACTTTGTGATCTTGATCAGATACACATTGACTGTGTATGGCATTTTATACTATCTTAATACATCTCTCTCATGTAGTTGATCAGATACCTGAGAACTTAGTCTTGCTCTCCCAAAGAAAGTTACATGGAGATTCCTTCTAAGGCATAGTCTCGGAAGCTGCTTCGTTATGCTATTCAAGGTTCCACTTTCGGAATAACCTTCATCATCTCACAAACATACCACTGAGCTGAAGGGAGAATGATAAGATGGAGAGAAGAGAAAACTAGAGGAAGGAGCATTATAA*TATGGTTAACTAATGTTTTAAAGAAAAAAGTCTAAAAAAAAAATCAAAAAGCTCTCCAAAAAACGTTCTCTGATCGAAACTTATTTAAATCTCCGTCACCGACTCCGGTGGTTCGCCGCTACCGGAGTCGCCCTTCTCCTTTCCTATTTTTCTTTTTTGCTTCTCTTCCATCTCCTAGCTACCTTCCGATATGCTTGTTCTTGTGGGGTTCGCTCTTCCAAGCCCTAGATCCGGCCAGATCTGAGGTACGGCAGTTGCAGGTTCCTGGAGGCACGGCGAGGAGGTGAGGCAGGTGAAGGAGGTTGAGTCGGCTCTTAGGGTGGGGAAAGATTGGTTCGGCGGTTTTGTAGATCTAGATCTGCTTGTTCGGTCGTCTTTTCCAGAAGTGGCAGCACGTGAGCTAAGGAGCTTCCTCTTCTCGGATCTGTTCCTCGGGTTTGTAGTCGGAGTGGGGTGGTGGTTCGTTTGCGGTATCCTCGCCTTGGCGTCTTGGCTATGGAGCTTCTCTTGACTCCACCCAAGGTGGGTGTCGGGGTCTATTCTCCGGTGGCGCGTCTCAGTGGCTACCTTGGTTTCGTGTGTTGGCTGGTGTGTCTCTGTCGACGGCGCGTGGGGGGTTCTCTGGCTGGTAAAGGCACGTGGAGTCGTCTATGGCTCTAGCGTCGGCTAGTCGAAGACCTTCCTCTGCCTTTGGATTTCTTCAGTTCTCGGTTTCTTCGTGTAGGAAGACCTGATTGAGATTTTCGAGGCTTCTCTTCAGGTTTACTTTGGGTCTACTGGCCTCGGTTCCGTGCCTCTGACCTGGTACTTTGCAGGCACGGTCTCGGCGGTTGGAAGATGATTCGTTTCTCTGGTTCTCAGAGGCGTAACGTGCGGTTGAGTTTGGGTTTCTACGGTCATGAGGAGACCTTCACTTCACCGGTGGCGGCGTTGTTGAGTGTTGCCATCCCAGTGGCGATTTTGATCGTCAGGTTACTCTCTTTCTCTTCCATTGTAGGTCCCGGAGTTTTCTTATCAGTGGTGCGGCTGTGACTTCAACCCGCTTTTGTATAGCTTTTTGAGAGTAGTCGCTTCCTTGCTGACTTTGTGTCGGTCTGTGGAGATGTTGTGTAAGCCTGGTGGTCTCGTTACTATTGTTGCGGCATCTGCGGGTTTCTTGGTTGTGGTTTGCTGTTTAGTTTGTTGAGCTGTAGGAGGATGTAGTAAGGCTTCGGTGTGTGGAGATGCGGCTTTCCTTTATTTGTGGGCTCCTGCTATTAAGCTTCCTTGTTTCTGGTTCTGAGAGGAGATGCGACTCCTCTTTGTTTGTGAGTTCCTGCTATTAAGCTCGGGTGATCGTTTCTGGTTCCGAGTGGAGATGCGACTCCTTCTTCTTGTGAGTTCCTGCTATTAAGCTCAGGTGATCGTTATTCTGAAGTGGAGATGCGGCTCTTCTTTGCGAGTGAGCTCCTGCTATTAAGCCCCGTTAGCTTGTGTTGTCTTGGCGTTAGTCCCATCTTGGTCCTTCGTTTTTGCTGCCATTTGAATCAGCTTCCTATGTGTTCGCAATCGGTTAGCACCCTCCTAGTGTTCTTGTATGTGTTAGCCTTGGTCGAACCTCTTCTCCGGGAATACAGTCTTTATCGCTAATTCTCTAGGTGGATGCTTACAAGTTGAAGTTTGGAGAATTACTGATTATCGTTTGTTTCCATGTTTTTATCTTTTTTTGCCGTGGAATCAATTATCCTAGGTTCTACAATTTGTTAACAACAGTCTGTAAAACCTCTTGTTCATTTTTCGAAATGATATTTACATTTTTAGCAAAAAAAAATATGGTTAACTA*TCAGCAAATATAAATGCGAGAATATCTTGAGTAATCCATATTGATTATTATATGCTTAATTACGGACAAAAATATATGATATAAATGCGGATTTTTTAAAGTAATCCACATTAACTGAATTATAAGAGAGATGCAATATTTTAATTTTGGAGTAAATTATAAGAGCGTTGTAAAATTGTGAAATTTCATGCAAGAAGATTAACTAAAACAAACGAAATCGGTCGAAAACAAAACAGTTCTTATTTGTTTTCACTTTTATATTGAACCATAATATCAATTGTATATAATATAGCGAGAATATATTTTGGAGGATTAAAACAAAAGATTGGGAGGATATAAGATTTATGAGAATATATCGCATATGACAGAATTTGAAGAATTTTTAGATGAATAACTAAATTACAATGATACAAAAAGTAATATAACATACAAAGTAAGAGCATTTTCAAAATAGCAGTCATGTTTTAATAGTGTCGATGATCATAAAAGGAAAATTGACTTTCTAAAAGGTTTGAGAAGTTATGAGAAAAGCAAAGTAGTTGGAAAAAAAACTTATATAGTCAAAATTAAACGGAAATTATATCAACAAAAAGGAAAGTAACAACAAAACCTAAATAAATTAGGTTTCAAAGATCCTCCTAGACTAAGAAATTGGTATTGTACCCCTATAAATACCCCGCCCGTATTAAACTCTACAATTCATCACTGCAATCAAACAGAACAAAGCAAAAACCCTAAGAAACTATAGATATCGTTTTTCATTATGAGATCCAGAGGCGACTTGTATTTCAGGCATGAGATCGACCGTAACCCTTCAGCCTCGGATGCTGGCACATTCAAAGCATGCGCAACCATGGTCACATGGCACGAGGACATGGAAGATTCTGAAACAACATTCTCGTTTACGTTGTCGGCGGAGGACGTCATCGAACGGCAAGGACTTGCCAACAAAATACAAGAACTAGATGAGCTATTCATGGAAGCTGCTTTCCCTCAAGAAGATAATTTGCTTTTGTTGACCCAAGAAGCCTATCATTGTTTCATTGAAGTGATTTCCTCAAGTGATTATAGCAGAGATTGTGCTGTGTCTCTGTGGTTTACTTTTCGTGTTTGGCGTTCTCCTCCTCCTCTTCCTCCTTCAGTTGATGAATTTGAGGATGATGATGATGATGATGATGAGGCGACCAATAGTAATATTCCAATCAGGGCAGCAAGCAAGCTCGCCATCAAGTCCTTAACCAAGAAAATATACAACAAATGTGACTCTCTTGCCATTGACAGTTGCACTATTTGTTTGGAAGAGTTTAAGAGTGGAGTCAATGTTGTCGAGTTACCCTGTGGACATGAATTTGATGATGCATGTATCGGACACTGGTTCGAGACCAATCACATTTGTCCATTGTGTCGTTTCGAGTTGCCTCGTGAGCATCATTGA

>MIZ *BrLINE1-RUP*

CATCACCATCATCAAGTCATGATTATTCCTCTGTCAAGGACTTTCCATCTTCAAAACGAATGAAGTCAGAATCTGACATTTTTTCTCCTTTTATTTGAAATACATAGAGCCAAGGCCTTAGCTAGCATTGTCTGTTAATATTCCACAACTAAGTTTTTTTGTTCATGCCTTTTTGTTTTGTGGTCAATACAAAGACTGTGTTTCTGGTTTTATTATCTTGTAAGGACTCAACTACTTCCTCCAGATTCTGGCGAGGTTTTGGTGTACTACACATTGACTGTGTATGCCATTTCTCTCATGTAGATGATCAGATACCTGAGAACTTTGTGATCTTGATCAGATACACATTGACTGTGTATGGCATTTTATACTATCTTAATACATCTCTCTCATGTAGTTGATCAGATACCTGAGAACTTAGTCTTGCTCTCCCAAAGAAAGTTACATGGAGATTCCTTCTAAGGCATAGTCTCGGAAGCTGCTTCGTTATGCTATTCAAGGTTCCACTTTCGGAATAACCTTCATCATCTCACAAACATACCACTGAGCTGAAGGGAGAATGATAAGATGGAGAGAAGAGAAAACTAGAGGAAGGAGCATTATAA*TATGGTTAACTAATGTTTTAAAGAAAAAAGTCTAAAAAAAAAATCAAAAAGCTCTCCAAAAAACGTTCTCTGATCGAAACTTATTTAAATCTCCGTCACCGACTCCGGTGGTTCGCCGCTACCGGAGTCGCCCTTCTCCTTTCCTATTTTTCTTTTTTGCTTCTCTTCCATCTCCTAGCTACCTTCCGATATGCTTGTTCTTGTGGGGTTCGCTCTTCCAAGCCCTAGATCCGGCCAGATCTGAGGTACGGCAGTTGCAGGTTCCTGGAGGCACGGCGAGGAGGTGAGGCAGGTGAAGGAGGTTGAGTCGGCTCTTAGGGTGGGGAAAGATTGGTTCGGCGGTTTTGTAGATCTAGATCTGCTTGTTCGGTCGTCTTTTCCAGAAGTGGCAGCACGTGAGCTAAGGAGCTTCCTCTTCTCGGATCTGTTCCTCGGGTTTGTAGTCGGAGTGGGGTGGTGGTTCGTTTGCGGTATCCTCGCCTTGGCGTCTTGGCTATGGAGCTTCTCTTGACTCCACCCAAGGTGGGTGTCGGGGTCTATTCTCCGGTGGCGCGTCTCAGTGGCTACCTTGGTTTCGTGTGTTGGCTGGTGTGTCTCTGTCGACGGCGCGTGGGGGGTTCTCTGGCTGGTAAAGGCACGTGGAGTCGTCTATGGCTCTAGCGTCGGCTAGTCGAAGACCTTCCTCTGCCTTTGGATTTCTTCAGTTCTCGGTTTCTTCGTGTAGGAAGACCTGATTGAGATTTTCGAGGCTTCTCTTCAGGTTTACTTTGGGTCTACTGGCCTCGGTTCCGTGCCTCTGACCTGGTACTTTGCAGGCACGGTCTCGGCGGTTGGAAGATGATTCGTTTCTCTGGTTCTCAGAGGCGTAACGTGCGGTTGAGTTTGGGTTTCTACGGTCATGAGGAGACCTTCACTTCACCGGTGGCGGCGTTGTTGAGTGTTGCCATCCCAGTGGCGATTTTGATCGTCAGGTTACTCTCTTTCTCTTCCATTGTAGGTCCCGGAGTTTTCTTATCAGTGGTGCGGCTGTGACTTCAACCCGCTTTTGTATAGCTTTTTGAGAGTAGTCGCTTCCTTGCTGACTTTGTGTCGGTCTGTGGAGATGTTGTGTAAGCCTGGTGGTCTCGTTACTATTGTTGCGGCATCTGCGGGTTTCTTGGTTGTGGTTTGCTGTTTAGTTTGTTGAGCTGTAGGAGGATGTAGTAAGGCTTCGGTGTGTGGAGATGCGGCTTTCCTTTATTTGTGGGCTCCTGCTATTAAGCTTCCTTGTTTCTGGTTCTGAGAGGAGATGCGACTCCTCTTTGTTTGTGAGTTCCTGCTATTAAGCTCGGGTGATCGTTTCTGGTTCCGAGTGGAGATGCGACTCCTTCTTCTTGTGAGTTCCTGCTATTAAGCTCAGGTGATCGTTATTCTGAAGTGGAGATGCGGCTCTTCTTTGCGAGTGAGCTCCTGCTATTAAGCCCCGTTAGCTTGTGTTGTCTTGGCGTTAGTCCCATCTTGGTCCTTCGTTTTTGCTGCCATTTGAATCAGCTTCCTATGTGTTCGCAATCGGTTAGCACCCTCCTAGTGTTCTTGTATGTGTTAGCCTTGGTCGAACCTCTTCTCCGGGAATACAGTCTTTATCGCTAATTCTCTAGGTGGATGCTTACAAGTTGAAGTTTGGAGAATTACTGATTATCGTTTGTTTCCATGTTTTTATCTTTTTTTGCCGTGGAATCAATTATCCTAGGTTCTACAATTTGTTAACAACAGTCTGTAAAACCTCTTGTTCATTTTTCGAAATGATATTTACATTTTTAGCAAAAAAAAATATGGTTAACTA*TCAGCAAATATAAATGCGAGAATATCTTGAGTAATCCATATTGATTATTATATGCTTAATTACGGACAAAAATATATGATATAAATGCGGATTTTTTAAAGTAATCCACATTAACTGAATTATAAGAGAGATGCAATATTTTAATTTTGGAGTAAATTATAAGAGCGTTGTAAAATTGTGAAATTTCATGCAAGAAGATTAACTAAAACAAACGAAATCGGTCGAAAACAAAACAGTTCTTATTTGTTTTCACTTTTATATTGAACCATAATATCAATTGTATATAATATAGCGAGAATATATTTTGGAGGATTAAAACAAAAGATTGGGAGGATATAAGATTTATGAGAATATATCGCATATGACAGAATTTGAAGAATTTTTAGATGAATAACTAAATTACAATGATACAAAAAGTAATATAACATACAAAGTAAGAGCATTTTCAAAATAGCAGTCATGTTTTAATAGTGTCGATGATCATAAAAGGAAAATTGACTTTCTAAAAGGTTTGAGAAGTTATGAGAAAAGCAAAGTAGTTGGAAAAAAAACTTATATAGTCAAAATTAAACGGAAATTATATCAACAAAAAGGAAAGTAACAACAAAACCTAAATAAATTAGGTTTCAAAGATCCTCCTAGACTAAGAAATTGGTATTGTACCCCTATAAATACCCCGCCCGTATTAAACTCTACAATTCATCACTGCAATCAAACAGAACAAAGCAAAAACCCTAAGAAACTATAGATATCGTTTTTCATTATGAGATCCAGAGGCGACTTGTATTTCAGGCATGAGATCGACCGTAACCCTTCAGCCTCGGATGCTGGCACATTCAAAGCATGCGCAACCATGGTCACATGGCACGAGGACATGGAAGATTCTGAAACAACATTCTCGTTTACGTTGTCGGCGGAGGACGTCATCGAACGGCAAGGACTTGCCAACAAAATACAAGAACTAGATGAGCTATTCATGGAAGCTGCTTTCCCTCAAGAAGATAATTTGCTTTTGTTGACCCAAGAAGCCTATCATTGTTTCATTGAAGTGATTTCCTCAAGTGATTATAGCAGAGATTGTGCTGTGTCTCTGTGGTTTACTTTTCGTGTTTGGCGTTCTCCTCCTCCTCTTCCTCCTTCAGTTGATGAATTTGAGGATGATGATGATGATGATGATGAGGCGACCAATAGTAATATTCCAATCAGGGCAGCAAGCAAGCTCGCCATCAAGTCCTTAACCAAGAAAATATACAACAAATGTGACTCTCTTGCCATTGACAGTTGCACTATTTGTTTGGAAGAGTTTAAGAGTGGAGTCAATGTTGTCGAGTTACCCTGTGGACATGAATTTGATGATGCATGTATCGGACACTGGTTCGAGACCAATCACATTTGTCCATTGTGTCGTTTCGAGTTGCCTCGTGAGCATCATTGA

>OIA *BrLINE1-RUP*

CCTCATCATCATCAAGTCATGATGATTCCTCTGTCAAGGACTTTCCATCTTCAAAACGAATGAAGTCAGAATCTGACATTTTTTCTCCTTTCATTTGAAATACTTAGAGCCAAGACCTTAGCTAGCATTGCCTGTTAATATTCCACAACTAAGTTTTTCTGTTCATGCCTTTTTGTTTTGTGGTCAATACAAAGACTGTGTTTCTGGTTTTGTTATCTTGTAAGAACTCAACTACTTCCTCCAGATTCTGGCGAGGGTTTGGTGTACTACACATTGACTGTGTATGCCATTTCTCTCATGTAGTTGATCAGATACCTGAGAACTTAGTGATCTTGATCAGATACACATTGACTGTGTATGGCATTTTATACTATCTTAATACATCTCTCTAATGTAGTTGATCAGATACCTGAGAACTTAGTCTTGCTCTCCCAAAGAAAGTTAAATGGAGATTCCTTTTAAGGCATAGTCTCGGAAGCTGCTTCGTTATGCTATTCAAGGTTCCACTTTCGGAATAACCTTCATCATCACAAACATACCACTGAGCTGAAGGGAGAATGATAAGATGGAGAGAAGAGAAAACTAGAGGAAGGAGCATTATAATATGGTTAACTATCAGCAAAAGTATACAAATATAAATGCGAGAATATCCTGAGTAATCCATACTGATTATATTATATGCTTAATTATGTACAAAAATATATGATATAAATGCGGATTTTTTTAAGTAATCCACATTAACTGAATTATAAGAGCGATGCAATATTTTAATTTGGACTAAATTATAAGCGCGTTGTATAATTGTGATATTTCATGCAAGAATTTCTCTTAACTAAAACAAACGAAATCGGTGGAAAACAAAATAGTTCTTATTTGTTTCACTTTTATATTGAACCATAATATCAATTGTATATAATATAGAGAGAATATATTTTGGAGGAATTAAAACAAAAGATTGGGAGGATATAAGATTTATGAGAATATCTCGCATATTACAGAATTTGAAGAATTTTTAGATGAAGAACTAAATTACAATGATACAAAAAGTAATATAACATACAAAGTAAGAGCATTTTCAAAATAGCAATCATGTTTTAATAGTGTCGATGATCATAAAAGGAAAATTGACTTTCTAAAACGTTTGAGAAGTTATTAGAAAAGCAAAGTAGTTGGAAAAAAAACTTATATAGCCAAAATTAAACGGAAATTATATCAACAAAGAGGAAAGTAACAACAAAACCTAAATAAATTAGGTTCCAAAGATCCTCCTAGACACAGAAATTGGTATGATCTTGTACCCCTATAAATACCCCGCCCATATTAAACTCTACAATAAATTCATCACTGCAATCAAACCGAACAAAGCAAAAACCCTAAGAAACTATAGATATCGTTTTTCATTATGAGATCCAGAGGCGACTTGTATTTCAGGCATGAGATCGACCATAACCCTCCAGCCTCGGATGCTGGCACAATCAAAGCATGCGCAACCATGGTCACGTGGCACGAGGACACGGAAGATTCTGAAACAACATTCTCGTTTACGTTGTCGGCGGATGACGTCATCGAACGCCAAGGACTTGCCAACAAAATACAAGAACTAGATGAGCTATTCATGGAAGCTGCTTTCCCTCAAGAAGATAATTTGCTTTTGTTGACCCAAGAAGCCTATCATTGTTTCATTGAAGTGATTTCCTCAAGTGATTATAGCAGAGATTGTGCTGTGTCTCTGTGGTTTACTTTTCGTGTTTGGCGTTCTCCTCCTCCTCTTCTTCCTTCAGTTGATGAATTTGAGGATGATGATGATGATGAGTCGACCAATAGTAATATTCCAATTAGGGCAGCAAGCAAGCTCGCCGTCAAGTGCTTAACCAAGAAAATATACAACAAATGTGACTCTCTCGCCATTGACAGTTGCACTATTTGTTTGGAAGAGTTTAAGAGTGGAGTCAATGTTGTCGAGTTACCCTGTGGACATGAATTTGATGATGCATGTATCGGACACTGGTTCGAGACCAATCACATTTGTCCATTGTGTCGTTTCGAGTTGCCTCGTGAGCATCATTGA

>OIB *BrLINE1-RUP*

CCTCATCATCATCAAGTCATGATGATTCCTCTGTCAAGGACTTTCCATCTTCAAAACGAATGAAGTCAGAATCTAACATTTTTTCTCCTTTCATTTGAAATACTTAGAGCCAAGACCTTAGCTAGCATTGTCTGTTAATATTCCACATCTAAGTTTTTTTGTTCATGCCTTTTTGTTTTGTGGTCAATACAAAGACTGTGTTTCTGGTTTTTTATCTTGTAAGAACTCAACTACTTCCTCCAGATTCTGGCGAGGTTTTGGTGTACTACATATTGACTGTGTATGCCATTTCTCTCATGTAGTTGATCAGATACCTGAGAACTTAGTGATCTTGATCAGATACACATTGACTGTGTATGGCATTTTATACTATCTTAATACATCTCTCTCATGTAGTTGATCAGATACCTGAGAACTTAGTCTTGCTCTCCCAAAGAAAGTTACATGGAGATTCCTTTTAAGGCATAGTCTCGGAAGCTGCTTCGTTATGCTATTCAAGGTTCCACTTTCGGAATAACCTTCATCATCACAAACATACCACTGAGCTGAAGGGAGAATGATAAGATGGAGAGAAGAGAAAACTAGAGGAAGGAGCATTATAATATGGTTAACTATCAGCAAATATAAATGCGAGAATATCTTGAGTAATCCATATTGATTATTATATGCTTAATTATGGACAAAAATATATGATATAAATGCGGATTTTTTTAAGTTATCCACATTAACTGAATTATAAGAGCGATGCAATATTTTAGTTTTGGACTAAATGATAAGAGCGTTTAAAATTGTGAAATTTCATGCAAGAAGATTAACTAAAACAAACGAAATCGGTGGAAAACAAAACAGTTCTTATTTGTTTTCACTTTTATATTGAACCATAATATCAATTGTATATAATATAGAGATAATATATTTTGGAGGAATTAAAACAAAAGATTGGGAGGATATAAGATTTATGAGAATATCTCGCATATTACAGAATTTGAAGAATTTTTAGATGAATAACTAAATTACAATGATACAAAAAGTAATATAACATACCAAGTAAGAGCGTTTTCAAAATAGCAACCATGTTTTAATAGTGTCGATGATCATAAAAGGAAAATTGACTTTCTAAAAGGTTTGAGAAGTTATGAGAAAAGCAAAGTAGTTGGAAAAAAAACTTATATAGTCAAAATTAAACGGAAATTATATCAACAAAAAGGAAAGTAACAACAAAACCTAAATAAATTAGGTTTCAAAGATCCTCCTAGACTAAGAAATTGGTATTGTACCCCTATAAATACCCCGCCCGTATTAAACTCTACAATTCATCACTGCAATCAAACAGAACAAAGCAAAAACCCTAAGAAACTATAGATATCGTTTTTTCATTATGAGATCCAGAGGCGACTTGTATTTCAGGCATGAGATCGACCGTAACCCTTCAGCCTCGGATGCTGGCACATTCAAAGCATGCGCAACCATGGTCACATGGCACGAGGACATGGAAGATTCTGAAACAACATTCTCGTTTACGTTGTCGGCGGAGGACGTCATCGAACGGCAAGGACTTGCCAACAAAATACAAGAACTAGATGAGCTATTCATGGAAGCTGCTTTCCCTCAAGAAGATAATTTGCTTTTGTTGACCCAAGAAGCATATCATTGTTTCATTGAAGTGATTTCCTCAAGTGATTATAGCAGAGATTGTGCTGTGTCTCTGTGGTTTACTTTTCGTGTTTGGCGTTCTCCTCCTCCTCTTCCTCCTTCAGTTGATGAATTTGAGGATGATGATGATGATGATGAGGCGACCAATAGTAATATTCCAATCAGGGCAGCAAGCAAGCTCGCCGTCAAGTCCTTAACCAAGAAAATATACAACAAATGTGACTCTCTCGCCATTGACAGTTGCACTATTTGTTTGGAAGAGTTTAAGAGTGGAGTCAATGTTGTCGAGTTACCCTGTGGACATGAATTTGATGATGCATGTATCGGACACTGGTTCGAGACCAATCACATTTGTCCATTGTGTCGTTTCGAGTTGGCTCGTGAGCATCATTGA

>OIC *BrLINE1-RUP*

CCTCATCATCATCAAGTCATGATGATTCCTCTGTCAAGGACTTTCCATCTTCAAAACGAATGAAGTCAGAATCTAACATTTTTTCTCCTTTCATTTGAAATACTTAGAGCCAAGACCTTAGCTAGCATTGTCTGTTAATATTCCACATCTAAGTTTTTTTGTTCATGCCTTTTTGTTTTGTGGTCAATACAAAGACTGTGTTTCTGGTTTTTTATCTTGTAAGAACTCAACTACTTCCTCCAGATTCTGGCGAGGTTTTGGTGTACTACACATTGACTGTGTATGCCATTTCTCTCATGTAGTTGATCAGATACCTGAGAACTTAGTGATCTTGATCAGATACACATTGACTGTGTATGGCATTTTATACTATCTTAATACATCTCTCTCATGTAGTTGATCAGATACCTGAGAACTTAGTCTTGCTCTCCCAAAGAAAGTTACATGGAGATTCCTTTTAAGGCATAGTCTCGGAAGCTGCTTCGTTATGCTATTCAAGGTTCCACTTTCGGAATAACCTTCATCATCACAAACATACCACTGAGCTGAAGGGAGAATGATAAGATGGAGAGAAGAGAAAACTAGAGGAAGGAGCATTATAATATGGTTAACTATCAGCAAATATAAATGCGAGAATATCTTGAGTAATCCATATTGATTATTATATGCTTAATTATGGACAAAAATATATGATATAAATGCGGATTTTTTTAAGTTATCCACATTAACTGAATTATAAGAGCGATGCAATATTTTAGTTTTGGACTAAATGATAAGAGCGTTTAAAATTGTGAAATTTCATGCAAGAAGATTAACTAAAACAAACGAAATCGGTGGAAAACAAAACAGTTCTTATTTGTTTTCACTTTTATATTGAACCATAATATCAATTGTATATAATATAGAGATAATATATTTTGGAGGAATTAAAACAAAAGATTGGGAGGATATAAGATTTATGAGAATATCTCGCATATTACAGAATTTGAAGAATTTTTAGATGAATAACTAAATTACAATGATACAAAAAGTAATATAACATACCAAGTAAGAGCGTTTTCAAAATAGCAACCATGTTTTAATAGTGTCGATGATCATAAAAGGAAAATTGACTTTCTAAAAGGTTTGAGAAGTTATGAGAAAAGCAAAGTAGTTGGAAAAAAAACTTATATAGTCAAAATTAAACGGAAATTATATCAACAAAAAGGAAAGTAACAACAAAACCTAAATAAATTAGGTTTCAAAGATCCTCCTAGACTAAGAAATTGGTATTGTACCCCTATAAATACCCCGCCCGTATTAAACTCTACAATTCATCACTGCAATCAAACAGAACAAAGCAAAAACCCTAAGAAACTATAGATATCGTTTTTCATTATGAGATCCAGAGGCGACTTGTATTTCAGGCATGAGATCGACCGTAACCCTTCAGCCTCGGATGCTGGCACATTCAAAGCATGCGCAACCATGGTCACATGGCACGAGGACATGGAAGATTCTGAAACAACATTCTCGTTTACGTTGTCGGCGGAGGACGTCATCGAACGGCAAGGACTTGCCAACAAAATACAAGAACTAGATGAGCTATTCATGGAAGCTGCTTTCCCTCAAGAAGATAATTTGCTTTTGTTGACCCAAGAAGCATATCATTGTTTCATTGAAGTGATTTCCTCAAGTGATTATAGCAGAGATTGTGCTGTGTCTCTGTGGTTTACTTTTCGTGTTTGGCGTTCTCCTCCTCCTCTTCCTCCTTCAGTTGATGAATTTGAGGATGATGATGATGATGATGAGGCGACCAATAGTAATATTCCAATCAGGGCAGCAAGCAAGCTCGCCGTCAAGTCCTTAACCAAGAAAATATACAACAAATGTGACTCTCTCGCCATTGACAGTTGCACTATTTGTTTGGAAGAGTTTAAGAGTGGAGTCAATGTTGTCGAGTTACCCTGTGGACATGAATTTGATGATGCATGTATCGGACACTGGTTCGAGACCAATCACATTTGTCCATTGTGTCGTTTCGAGTTGGCTCGTGAGCATCATTGA

>PCA *BrLINE1-RUP*

CATCACCATCATCAAGTCATGATTATTCCTCTGTCAAGGACTTTCCATCTTCAAAACGAATGAAGTCAGAATCTGACATTTTTTCTCCTTTTATTTGAAATACATAGAGCCAAGGCCTTAGCTAGCATTGTCTGTTAATATTCCACAACTAAGTTTTTTTGTTCATGCCTTTTTGTTTTGTGGTCAATACAAAGACTGTGTTTCTGGTTTTATTATCTTGTAAGGACTCAACTACTTCCTCCAGATTCTGGCGAGGTTTTGGTGTACTACACATTGACTGTGTATGCCATTTCTCTCATGTAGATGATCAGATACCTGAGAACTTTGTGATCTTGATCAGATACACATTGACTGTGTATGGCATTTTATACTATCTTAATACATCTCTCTCATGTAGTTGATCAGATACCTGAGAACTTAGTCTTGCTCTCCCAAAGAAAGTTACATGGAGATTCCTTCTAAGGCATAGTCTCGGAAGCTGCTTCGTTATGCTATTCAAGGTTCCACTTTCGGAATAACCTTCATCATCTCACAAACATACCACTGAGCTGAAGGGAGAATGATAAGATGGAGAGAAGAGAAAACTAGAGGAAGGAGCATTATAA*TATGGTTAACTAATGTTTTAAAGAAAAAAGTCTAAAAAAAAAATCAAAAAGCTCTCCAAAAAACGTTCTCTGATCGAAACTTATTTAAATCTCCGTCACCGACTCCGGTGGTTCGCCGCTACCGGAGTCGCCCTTCTCCTTTCCTATTTTTCTTTTTTGCTTCTCTTCCATCTCCTAGCTACCTTCCGATATGCTTGTTCTTGTGGGGTTCGCTCTTCCAAGCCCTAGATCCGGCCAGATCTGAGGTACGGCAGTTGCAGGTTCCTGGAGGCACGGCGAGGAGGTGAGGCAGGTGAAGGAGGTTGAGTCGGCTCTTAGGGTGGGGAAAGATTGGTTCGGCGGTTTTGTAGATCTAGATCTGCTTGTTCGGTCGTCTTTTCCAGAAGTGGCAGCACGTGAGCTAAGGAGCTTCCTCTTCTCGGATCTGTTCCTCGGGTTTGTAGTCGGAGTGGGGTGGTGGTTCGTTTGCGGTATCCTCGCCTTGGCGTCTTGGCTATGGAGCTTCTCTTGACTCCACCCAAGGTGGGTGTCGGGGTCTATTCTCCGGTGGCGCGTCTCAGTGGCTACCTTGGTTTCGTGTGTTGGCTGGTGTGTCTCTGTCGACGGCGCGTGGGGGGTTCTCTGGCTGGTAAAGGCACGTGGAGTCGTCTATGGCTCTAGCGTCGGCTAGTCGAAGACCTTCCTCTGCCTTTGGATTTCTTCAGTTCTCGGTTTCTTCGTGTAGGAAGACCTGATTGAGATTTTCGAGGCTTCTCTTCAGGTTTACTTTGGGTCTACTGGCCTCGGTTCCGTGCCTCTGACCTGGTACTTTGCAGGCACGGTCTCGGCGGTTGGAAGATGATTCGTTTCTCTGGTTCTCAGAGGCGTAACGTGCGGTTGAGTTTGGGTTTCTACGGTCATGAGGAGACCTTCACTTCACCGGTGGCGGCGTTGTTGAGTGTTGCCATCCCAGTGGCGATTTTGATCGTCAGGTTACTCTCTTTCTCTTCCATTGTAGGTCCCGGAGTTTTCTTATCAGTGGTGCGGCTGTGACTTCAACCCGCTTTTGTATAGCTTTTTGAGAGTAGTCGCTTCCTTGCTGACTTTGTGTCGGTCTGTGGAGATGTTGTGTAAGCCTGGTGGTCTCGTTACTATTGTTGCGGCATCTGCGGGTTTCTTGGTTGTGGTTTGCTGTTTAGTTTGTTGAGCTGTAGGAGGATGTAGTAAGGCTTCGGTGTGTGGAGATGCGGCTTTCCTTTATTTGTGGGCTCCTGCTATTAAGCTTCCTTGTTTCTGGTTCTGAGAGGAGATGCGACTCCTCTTTGTTTGTGAGTTCCTGCTATTAAGCTCGGGTGATCGTTTCTGGTTCCGAGTGGAGATGCGACTCCTTCTTCTTGTGAGTTCCTGCTATTAAGCTCAGGTGATCGTTATTCTGAAGTGGAGATGCGGCTCTTCTTTGCGAGTGAGCTCCTGCTATTAAGCCCCGTTAGCTTGTGTTGTCTTGGCGTTAGTCCCATCTTGGTCCTTCGTTTTTGCTGCCATTTGAATCAGCTTCCTATGTGTTCGCAATCGGTTAGCACCCTCCTAGTGTTCTTGTATGTGTTAGCCTTGGTCGAACCTCTTCTCCGGGAATACAGTCTTTATCGCTAATTCTCTAGGTGGATGCTTACAAGTTGAAGTTTGGAGAATTACTGATTATCGTTTGTTTCCATGTTTTTATCTTTTTTTGCCGTGGAATCAATTATCCTAGGTTCTACAATTTGTTAACAACAGTCTGTAAAACCTCTTGTTCATTTTTCGAAATGATATTTACATTTTTAGCAAAAAAAAATATGGTTAACTA*TCAGCAAATATAAATGCGAGAATATCTTGAGTAATCCATATTGATTATTATATGCTTAATTACGGACAAAAATATATGATATAAATGCGGATTTTTTAAAGTAATCCACATTAACTGAATTATAAGAGAGATGCAATATTTTAATTTTGGAGTAAATTATAAGAGCGTTGTAAAATTGTGAAATTTCATGCAAGAAGATTAACTAAAACAAACGAAATCGGTCGAAAACAAAACAGTTCTTATTTGTTTTCACTTTTATATTGAACCATAATATCAATTGTATATAATATAGCGAGAATATATTTTGGAGGATTAAAACAAAAGATTGGGAGGATATAAGATTTATGAGAATATATCGCATATGACAGAATTTGAAGAATTTTTAGATGAATAACTAAATTACAATGATACAAAAAGTAATATAACATACAAAGTAAGAGCATTTTCAAAATAGCAGTCATGTTTTAATAGTGTCGATGATCATAAAAGGAAAATTGACTTTCTAAAAGGTTTGAGAAGTTATGAGAAAAGCAAAGTAGTTGGAAAAAAAACTTATATAGTCAAAATTAAACGGAAATTATATCAACAAAAAGGAAAGTAACAACAAAACCTAAATAAATTAGGTTTCAAAGATCCTCCTAGACTAAGAAATTGGTATTGTACCCCTATAAATACCCCGCCCGTATTAAACTCTACAATTCATCACTGCAATCAAACAGAACAAAGCAAAAACCCTAAGAAACTATAGATATCGTTTTTCATTATGAGATCCAGAGGCGACTTGTATTTCAGGCATGAGATCGACCGTAACCCTTCAGCCTCGGATGCTGGCACATTCAAAGCATGCGCAACCATGGTCACATGGCACGAGGACATGGAAGATTCTGAAACAACATTCTCGTTTACGTTGTCGGCGGAGGACGTCATCGAACGGCAAGGACTTGCCAACAAAATACAAGAACTAGATGAGCTATTCATGGAAGCTGCTTTCCCTCAAGAAGATAATTTGCTTTTGTTGACCCAAGAAGCCTATCATTGTTTCATTGAAGTGATTTCCTCAAGTGATTATAGCAGAGATTGTGCTGTGTCTCTGTGGTTTACTTTTCGTGTTTGGCGTTCTCCTCCTCCTCTTCCTCCTTCAGTTGATGAATTTGAGGATGATGATGATGATGATGATGAGGCGACCAATAGTAATATTCCAATCAGGGCAGCAAGCAAGCTCGCCATCAAGTCCTTAACCAAGAAAATATACAACAAATGTGACTCTCTTGCCATTGACAGTTGCACTATTTGTTTGGAAGAGTTTAAGAGTGGAGTCAATGTTGTCGAGTTACCCTGTGGACATGAATTTGATGATGCATGTATCGGACACTGGTTCGAGACCAATCACATTTGTCCATTGTGTCGTTTCGAGTTGCCTCGTGAGCATCATTGA

>PCB *BrLINE1-RUP*

CCTCATCATCATCAAGTCATGATGATTCCTCTGTCAAGGACTTTCCATCTTCAAAACGAATGAAGTCAGAATCTGACATTTTTTCTCCTTTCATTTGAAATACTTAGAGCCAAGACCTTAGCTAGCATTGCCTGTTAATATTCCACAACTAAGTTTTTCTGTTCATGCCTTTTTGTTTTGTGGTCAATACAAAGACTGTGTTTCTGGTTTTGTTATCTTGTAAGAACTCAACTACTTCCTCCAGATTCTGGCGAGGGTTTGGTGTACTACACATTGACTGTGTATGCCATTTCCTCATGTAGTTGATCAGATACCTGAGAACTTAGTGATCTTGATCAGATACACATTGACTGTGTATGGCATTTTATACTATCTTAATACATCTCTCTCATGTAGTTGATCAGATACCTGAGAACTTAGTCTTGCTCTCCCAAAGAAAGTTACATGGAGATTCCTTTTAAGGCATAGTCTCGGAAGCTGCTTCGTTATGCTATTCAAGGTTCCACTTTCGGAATAACCTTCATCATCACAAACATACCACTGAGCTGAAGGGAGAATGATAAGATGGAGAGAAGAGAAAACTAGAGGAAGGAGCATTATAATATGGTTAACTATCAGCAAAAGTATACAAATATAAATGCGAGAATATCCTGAGTAATCCATATTGATATTATATTATACTAGGTCTTTGTCCGCGCTACGCGCGGATAGTATTTTGATTTTTTTCATATTTTTGTACTATTACGTCAATTATTTAGTTTTATAAAATGTTATGTTTTTACTGTAAATTTGGAATTAAAAATCTAATTTTTTCTTACTGTAAAAATAAGTTAATTTTTTTGAATCTTACCACAACACATTATACACGAAGAGAATATATAGTTGGTCTTTATATTCTTATTTGGTGTAATATTGAAAATTTTGATGGTTTGTAGTTTTTTTAAATTATATATTTTAGGATTGATTTTCGTGAGTATATTCTATAATTGTCTAAAAAAATTGTTTGTCCCATATAGACATCCACATAAATATGGACTTCTGATAAATTTGTTCATTGGGATATTTAGTTTCACTTTCAACTTTATTCTCTTTTTGGCACCCTCATGCTAGCTTGTGGGGCTAGTCAACTTGGTGCAAAAGGGTTTACAAAAGCTGATCGAGATGCGCGTGATCGGACACCTTTTGCTAGGCTATTCATACGGAATTTTAAAGATCTAGGAATATAAGCAATAGAAAGTTCAGAAAATTCTTTGGATACAACCTGTATTTCGTCGAGCTCCGAGTCCAACGCAGGCTAATCTTTCTCCTTTTGAATGAGTATAACCAGTTGTTCACAGTCGATTGAAAGATCATCTCTCTGTATCCAAACTTCAGTATCACTTGCATTGCCCATAGTAAACCTTCAACTTCCGCTTGCAGTGGTGATTTGGTGCGTGTATATCGGCCCTTGCTCCAAACAGCATTGGAAAGTCACCATCCATTAAAACAAAGCCAAGTCCAGATATGTCTCTTTCATTTATCCAGGATGTCTAGCAGGAGCGTTTCTTTGCGGAGGAACAGTTGTGTTTGTTACCTCAACTCCCATATCAATCTCCATAATCTCGTCGATACATTGAGCTATCCTCCAACACTCTGCTTCAATTTTATTCGCTAATAGGAAAGACATAATTCCTACGACACTATTAATGATTGTTTTTTTTGTAACACCGATACGGCAAACTTATGTTTTATTTAACAAAACTCTTATTTTAATTTTGTATTAAAGAATCAATCATATTTTATATTATCCATAATTTTAGTAAATTTGCTTATTTGTCATGTTTTTATTAGGTTTTAGCTAAATCATTGATTTATTATTTATTTTTAGCTAAGTTACTAATTTATTAATTTAAAAATACCCTTAATTAATATTATATATATATATTAAAAATGAATTTTATTTTAGTAATATTAAATTTATATTTTATATTAAAATTTAGTTAGTTTTTCTTATTTGTCATATTTTATTAGGTTTTAGACTTTTAGATAGTTATTGATTTATTATTAATTTCTAACTGATTCGCTAATGTGTTAATTAAAACAATACCCTTAATGAATTTTATATATAATTAAAAACGAATTTTATTTTAATCATATAAAATTTATATGTTATATTAATATTAAATATTTGATTCTAAAATAATATAAAAATTATAAAAAAAATTAAAAATAAGATAATTCTTATATATATTTTGTTGCTATCTGAAAAAAATATTTTTATTATAAATGTTAAAAAGATACAAAAAATTATAATTAAATATTATTCAAGAAAAAACATTTATATAAATATATTTTCTAAACTATTTCTAAGATATGAGTATTTTAAAAAATTTAACACGGGATGTTTAGAACACGGAATTATGCTTATGAGAGAGTTGCTCGGGAGTGGGCTAGGAGATGGGTCTAATGGGCTGCAAATTATTTTGAATTTTTTTTAATTGCAAGCCCATTTCGTGATGACCTGGCATATTGGTTGGTCCTGAATATTTGTGCACATGTGGATAGGCTTAGGAATCAATGATTTAGCTCCTTTTATATAGTAGGATGTTTAATTATGGACAAAAATATTTGATATAAATGCGGATTTTTGAAGTAATCCACATTAACTGAATTATAAGAGCGATGCAATATTTTAATTTTGGACTAAATTATAAGAGCGTTGTAAAATTGTGAAATTTCATGCAAGAAGATTAACTAAAACAAACGAAATCGGTGGAAAACAAAACAGTTCTTATTTGTTTTCACTTTTATATTGAACCATAATATCAATTGTATACTATAATATAGCGAGAATATATTTTGGAGGAATTAAAACAAAAGATTGGGAGGATATAAGATTTATGAGAATATCTCGCATATGACAGAATTTGAAGAATTTTTAGATGAATAACTAAATTACAATGATACAAAAAGTAATATAACATACAAAGCAAGAGCATTTTCAAAATAGCAATCATGTTTTAATAGTGTCGATGATCATAAAAGGAAAATGGACTTTCTAAAAGGTTTGAGAAGTTATTAGAAAAGCAAAGTAGTTGGAAAAAAAACTTGAAAATCCTCGTGTCACCAGTTCAAATCTGGTTCTTGGCATAGGGCAGAGGACTGAAAATCCTCGTGTCACCAGTTCAAATCTGGTTATTGGCATAGGATTGATTAATTTTGATAAGTTTATAGTCTTCAAATTAAACGTATCTTTAGTAAAAAAAGTGTTTGCATGGTCCAATCGTTTATCCCGAAAATTTCATATAGTCAAAATTAAACGGAAATTATATCAACAAAAGGGAAAGTAACAACAAAACCTAAATAAATTAGTATGATCTTGTTTCTAAAAAAACCCTAAGAAACTATAGATATCGTTTTTCATTATGAGATCTAGAGGCGACTTGTATTTCAGGCATGAGATCGACCGTAACCCTTAAGCCTCGGATGCTGGCACAATCAAAGCATGCGCAACCATGGTCACATGGCAGGAGGACATGGCAGATTTTGAAACAACATTCTCGTTTACGTTGTCGGCGGAGGACATCATCGAACGCCAAAGACTTGCCAACAAAATACAAGAACTAGATGAGTTATTCATGGAATCTGCTTTCCCTCGAGAAGATAATTTGCTTTTGTTGACCCAAGAAGCCTATCATTGTTTCATTAAATTGATTTCCTCACGTGATTATAGTAGAGATTGTGTTATGTCTCTGTGGTTTACTTTTCGTGTTTGGCGTACTCCTCTTCCTCCTTCAATTGATGAATGTGAGGATGATGATGATGATGAGGCGACCAATAGTAATATTCCAATCAGGACAGCAAGCAAGCTCGCGGTCAAGTCCTTAACCAAGAAAATATACAACAAAGGTGACTCTCTCGCCATTGACAGTTGCACTATTTATACCCGGTTGGAAGGGTTAATCACGACCTTTATATCCGGTCGGAAGGGTTAA

>TCA *BrLINE1-RUP*

CATCACCATCATCAAGTCATGATTATTCCTCTGTCAAGGACTTTCCATCTTCAAAACGAATGAAGTCAGAATCTGACATTTTTTCTCCTTTTATTTGAAATACATAGAGCCAAGGCCTTAGCTAGCATTGTCTGTTAATATTCCACAACTAAGTTTTTTTGTTCATGCCTTTTTGTTTTGTGGTCAATACAAAGACTGTGTTTCTGGTTTTATTATCTTGTAAGGACTCAACTACTTCCTCCAGATTCTGGCGAGGTTTTGGTGTACTACACATTGACTGTGTATGCCATTTCTCTCATGTAGATGATCAGATACCTGAGAACTTTGTGATCTTGATCAGATACACATTGACTGTGTATGGCATTTTATACTATCTTAATACATCTCTCTCATGTAGTTGATCAGATACCTGAGAACTTAGTCTTGCTCTCCCAAAGAAAGTTACATGGAGATTCCTTCTAAGGCATAGTCTCGGAAGCTGCTTCGTTATGCTATTCAAGGTTCCACTTTCGGAATAACCTTCATCATCTCACAAACATACCACTGAGCTGAAGGGAGAATGATAAGATGGAGAGAAGAGAAAACTAGAGGAAGGAGCATTATAA*TATGGTTAACTAATGTTTTAAAGAAAAAAGTCTAAAAAAAAAATCAAAAAGCTCTCCAAAAAACGTTCTCTGATCGAAACTTATTTAAATCTCCGTCACCGACTCCGGTGGTTCGCCGCTACCGGAGTCGCCCTTCTCCTTTCCTATTTTTCTTTTTTGCTTCTCTTCCATCTCCTAGCTACCTTCCGATATGCTTGTTCTTGTGGGGTTCGCTCTTCCAAGCCCTAGATCCGGCCAGATCTGAGGTACGGCAGTTGCAGGTTCCTGGAGGCACGGCGAGGAGGTGAGGCAGGTGAAGGAGGTTGAGTCGGCTCTTAGGGTGGGGAAAGATTGGTTCGGCGGTTTTGTAGATCTAGATCTGCTTGTTCGGTCGTCTTTTCCAGAAGTGGCAGCACGTGAGCTAAGGAGCTTCCTCTTCTCGGATCTGTTCCTCGGGTTTGTAGTCGGAGTGGGGTGGTGGTTCGTTTGCGGTATCCTCGCCTTGGCGTCTTGGCTATGGAGCTTCTCTTGACTCCACCCAAGGTGGGTGTCGGGGTCTATTCTCCGGTGGCGCGTCTCAGTGGCTACCTTGGTTTCGTGTGTTGGCTGGTGTGTCTCTGTCGACGGCGCGTGGGGGGTTCTCTGGCTGGTAAAGGCACGTGGAGTCGTCTATGGCTCTAGCGTCGGCTAGTCGAAGACCTTCCTCTGCCTTTGGATTTCTTCAGTTCTCGGTTTCTTCGTGTAGGAAGACCTGATTGAGATTTTCGAGGCTTCTCTTCAGGTTTACTTTGGGTCTACTGGCCTCGGTTCCGTGCCTCTGACCTGGTACTTTGCAGGCACGGTCTCGGCGGTTGGAAGATGATTCGTTTCTCTGGTTCTCAGAGGCGTAACGTGCGGTTGAGTTTGGGTTTCTACGGTCATGAGGAGACCTTCACTTCACCGGTGGCGGCGTTGTTGAGTGTTGCCATCCCAGTGGCGATTTTGATCGTCAGGTTACTCTCTTTCTCTTCCATTGTAGGTCCCGGAGTTTTCTTATCAGTGGTGCGGCTGTGACTTCAACCCGCTTTTGTATAGCTTTTTGAGAGTAGTCGCTTCCTTGCTGACTTTGTGTCGGTCTGTGGAGATGTTGTGTAAGCCTGGTGGTCTCGTTACTATTGTTGCGGCATCTGCGGGTTTCTTGGTTGTGGTTTGCTGTTTAGTTTGTTGAGCTGTAGGAGGATGTAGTAAGGCTTCGGTGTGTGGAGATGCGGCTTTCCTTTATTTGTGGGCTCCTGCTATTAAGCTTCCTTGTTTCTGGTTCTGAGAGGAGATGCGACTCCTCTTTGTTTGTGAGTTCCTGCTATTAAGCTCGGGTGATCGTTTCTGGTTCCGAGTGGAGATGCGACTCCTTCTTCTTGTGAGTTCCTGCTATTAAGCTCAGGTGATCGTTATTCTGAAGTGGAGATGCGGCTCTTCTTTGCGAGTGAGCTCCTGCTATTAAGCCCCGTTAGCTTGTGTTGTCTTGGCGTTAGTCCCATCTTGGTCCTTCGTTTTTGCTGCCATTTGAATCAGCTTCCTATGTGTTCGCAATCGGTTAGCACCCTCCTAGTGTTCTTGTATGTGTTAGCCTTGGTCGAACCTCTTCTCCGGGAATACAGTCTTTATCGCTAATTCTCTAGGTGGATGCTTACAAGTTGAAGTTTGGAGAATTACTGATTATCGTTTGTTTCCATGTTTTTATCTTTTTTTGCCGTGGAATCAATTATCCTAGGTTCTACAATTTGTTAACAACAGTCTGTAAAACCTCTTGTTCATTTTTCGAAATGATATTTACATTTTTAGCAAAAAAAAATATGGTTAACTA*TCAGCAAATATAAATGCGAGAATATCTTGAGTAATCCATATTGATTATTATATGCTTAATTACGGACAAAAATATATGATATAAATGCGGATTTTTTAAAGTAATCCACATTAACTGAATTATAAGAGAGATGCAATATTTTAATTTTGGAGTAAATTATAAGAGCGTTGTAAAATTGTGAAATTTCATGCAAGAAGATTAACTAAAACAAACGAAATCGGTCGAAAACAAAACAGTTCTTATTTGTTTTCACTTTTATATTGAACCATAATATCAATTGTATATAATATAGCGAGAATATATTTTGGAGGATTAAAACAAAAGATTGGGAGGATATAAGATTTATGAGAATATATCGCATATGACAGAATTTGAAGAATTTTTAGATGAATAACTAAATTACAATGATACAAAAAGTAATATAACATACAAAGTAAGAGCATTTTCAAAATAGCAGTCATGTTTTAATAGTGTCGATGATCATAAAAGGAAAATTGACTTTCTAAAAGGTTTGAGAAGTTATGAGAAAAGCAAAGTAGTTGGAAAAAAAACTTATATAGTCAAAATTAAACGGAAATTATATCAACAAAAAGGAAAGTAACAACAAAACCTAAATAAATTAGGTTTCAAAGATCCTCCTAGACTAAGAAATTGGTATTGTACCCCTATAAATACCCCGCCCGTATTAAACTCTACAATTCATCACTGCAATCAAACAGAACAAAGCAAAAACCCTAAGAAACTATAGATATCGTTTTTCATTATGAGATCCAGAGGCGACTTGTATTTCAGGCATGAGATCGACCGTAACCCTTCAGCCTCGGATGCTGGCACATTCAAAGCATGCGCAACCATGGTCACATGGCACGAGGACATGGAAGATTCTGAAACAACATTCTCGTTTACGTTGTCGGCGGAGGACGTCATCGAACGGCAAGGACTTGCCAACAAAATACAAGAACTAGATGAGCTATTCATGGAAGCTGCTTTCCCTCAAGAAGATAATTTGCTTTTGTTGACCCAAGAAGCCTATCATTGTTTCATTGAAGTGATTTCCTCAAGTGATTATAGCAGAGATTGTGCTGTGTCTCTGTGGTTTACTTTTCGTGTTTGGCGTTCTCCTCCTCCTCTTCCTCCTTCAGTTGATGAATTTGAGGATGATGATGATGATGATGATGAGGCGACCAATAGTAATATTCCAATCAGGGCAGCAAGCAAGCTCGCCATCAAGTCCTTAACCAAGAAAATATACAACAAATGTGACTCTCTTGCCATTGACAGTTGCACTATTTGTTTGGAAGAGTTTAAGAGTGGAGTCAATGTTGTCGAGTTACCCTGTGGACATGAATTTGATGATGCATGTATCGGACACTGGTTCGAGACCAATCACATTTGTCCATTGTGTCGTTTCGAGTTGCCTCGTGAGCATCATTGA

>TUA *BrLINE1-RUP*

CATCACCATCATCAAGTCATGATTATTCCTCTGTCAAGGACTTTCCATCTTCAAAACGAATGAAGTCAGAATCTGACATTTTTTCTCCTTTTATTTGAAATACATAGAGCCAAGGCCTTAGCTAGCATTGTCTGTTAATATTCCACAACTAAGTTTTTTTGTTCATGCCTTTTTGTTTTGTGGTCAATACAAAGACTGTGTTTCTGGTTTTATTATCTTGTAAGGACTCAACTACTTCCTCCAGATTCTGGCGAGGTTTTGGTGTACTACACATTGACTGTGTATGCCATTTCTCTCATGTAGATGATCAGATACCTGAGAACTTTGTGATCTTGATCAGATACACATTGACTGTGTATGGCATTTTATACTATCTTAATACATCTCTCTCATGTAGTTGATCAGATACCTGAGAACTTAGTCTTGCTCTCCCAAAGAAAGTTACATGGAGATTCCTTCTAAGGCATAGTCTCGGAAGCTGCTTCGTTATGCTATTCAAGGTTCCACTTTCGGAATAACCTTCATCATCTCACAAACATACCACTGAGCTGAAGGGAGAATGATAAGATGGAGAGAAGAGAAAACTAGAGGAAGGAGCATTATAA*TATGGTTAACTAATGTTTTAAAGAAAAAAGTCTAAAAAAAAAATCAAAAAGCTCTCCAAAAAACGTTCTCTGATCGAAACTTATTTAAATCTCCGTCACCGACTCCGGTGGTTCGCCGCTACCGGAGTCGCCCTTCTCCTTTCCTATTTTTCTTTTTTGCTTCTCTTCCATCTCCTAGCTACCTTCCGATATGCTTGTTCTTGTGGGGTTCGCTCTTCCAAGCCCTAGATCCGGCCAGATCTGAGGTACGGCAGTTGCAGGTTCCTGGAGGCACGGCGAGGAGGTGAGGCAGGTGAAGGAGGTTGAGTCGGCTCTTAGGGTGGGGAAAGATTGGTTCGGCGGTTTTGTAGATCTAGATCTGCTTGTTCGGTCGTCTTTTCCAGAAGTGGCAGCACGTGAGCTAAGGAGCTTCCTCTTCTCGGATCTGTTCCTCGGGTTTGTAGTCGGAGTGGGGTGGTGGTTCGTTTGCGGTATCCTCGCCTTGGCGTCTTGGCTATGGAGCTTCTCTTGACTCCACCCAAGGTGGGTGTCGGGGTCTATTCTCCGGTGGCGCGTCTCAGTGGCTACCTTGGTTTCGTGTGTTGGCTGGTGTGTCTCTGTCGACGGCGCGTGGGGGGTTCTCTGGCTGGTAAAGGCACGTGGAGTCGTCTATGGCTCTAGCGTCGGCTAGTCGAAGACCTTCCTCTGCCTTTGGATTTCTTCAGTTCTCGGTTTCTTCGTGTAGGAAGACCTGATTGAGATTTTCGAGGCTTCTCTTCAGGTTTACTTTGGGTCTACTGGCCTCGGTTCCGTGCCTCTGACCTGGTACTTTGCAGGCACGGTCTCGGCGGTTGGAAGATGATTCGTTTCTCTGGTTCTCAGAGGCGTAACGTGCGGTTGAGTTTGGGTTTCTACGGTCATGAGGAGACCTTCACTTCACCGGTGGCGGCGTTGTTGAGTGTTGCCATCCCAGTGGCGATTTTGATCGTCAGGTTACTCTCTTTCTCTTCCATTGTAGGTCCCGGAGTTTTCTTATCAGTGGTGCGGCTGTGACTTCAACCCGCTTTTGTATAGCTTTTTGAGAGTAGTCGCTTCCTTGCTGACTTTGTGTCGGTCTGTGGAGATGTTGTGTAAGCCTGGTGGTCTCGTTACTATTGTTGCGGCATCTGCGGGTTTCTTGGTTGTGGTTTGCTGTTTAGTTTGTTGAGCTGTAGGAGGATGTAGTAAGGCTTCGGTGTGTGGAGATGCGGCTTTCCTTTATTTGTGGGCTCCTGCTATTAAGCTTCCTTGTTTCTGGTTCTGAGAGGAGATGCGACTCCTCTTTGTTTGTGAGTTCCTGCTATTAAGCTCGGGTGATCGTTTCTGGTTCCGAGTGGAGATGCGACTCCTTCTTCTTGTGAGTTCCTGCTATTAAGCTCAGGTGATCGTTATTCTGAAGTGGAGATGCGGCTCTTCTTTGCGAGTGAGCTCCTGCTATTAAGCCCCGTTAGCTTGTGTTGTCTTGGCGTTAGTCCCATCTTGGTCCTTCGTTTTTGCTGCCATTTGAATCAGCTTCCTATGTGTTCGCAATCGGTTAGCACCCTCCTAGTGTTCTTGTATGTGTTAGCCTTGGTCGAACCTCTTCTCCGGGAATACAGTCTTTATCGCTAATTCTCTAGGTGGATGCTTACAAGTTGAAGTTTGGAGAATTACTGATTATCGTTTGTTTCCATGTTTTTATCTTTTTTTGCCGTGGAATCAATTATCCTAGGTTCTACAATTTGTTAACAACAGTCTGTAAAACCTCTTGTTCATTTTTCGAAATGATATTTACATTTTTAGCAAAAAAAAATATGGTTAACTA*TCAGCAAATATAAATGCGAGAATATCTTGAGTAATCCATATTGATTATTATATGCTTAATTACGGACAAAAATATATGATATAAATGCGGATTTTTTAAAGTAATCCACATTAACTGAATTATAAGAGAGATGCAATATTTTAATTTTGGAGTAAATTATAAGAGCGTTGTAAAATTGTGAAATTTCATGCAAGAAGATTAACTAAAACAAACGAAATCGGTCGAAAACAAAACAGTTCTTATTTGTTTTCACTTTTATATTGAACCATAATATCAATTGTATATAATATAGCGAGAATATATTTTGGAGGATTAAAACAAAAGATTGGGAGGATATAAGATTTATGAGAATATATCGCATATGACAGAATTTGAAGAATTTTTAGATGAATAACTAAATTACAATGATACAAAAAGTAATATAACATACAAAGTAAGAGCATTTTCAAAATAGCAGTCATGTTTTAATAGTGTCGATGATCATAAAAGGAAAATTGACTTTCTAAAAGGTTTGAGAAGTTATGAGAAAAGCAAAGTAGTTGGAAAAAAAACTTATATAGTCAAAATTAAACGGAAATTATATCAACAAAAAGGAAAGTAACAACAAAACCTAAATAAATTAGGTTTCAAAGATCCTCCTAGACTAAGAAATTGGTATTGTACCCCTATAAATACCCCGCCCGTATTAAACTCTACAATTCATCACTGCAATCAAACAGAACAAAGCAAAAACCCTAAGAAACTATAGATATCGTTTTTCATTATGAGATCCAGAGGCGACTTGTATTTCAGGCATGAGATCGACCGTAACCCTTCAGCCTCGGATGCTGGCACATTCAAAGCATGTGCAACCATGGTCACATGGCACGAGGACATGGAAGATTCTGAAACAACATTCTCGTTTACGTTGTCGGCGGAGGACGTCATCGAACGGCAAGGACTTGCCAACAAAATACAAGAACTAGATGAGCTATTCATGGAAGCTGCTTTCCCTCAAGAAGATAATTTGCTTTTGTTGACCCAAGAAGCCTATCATTGTTTCATTGAAGTGATTTCCTCAAGTGATTATAGCAGAGATTGTGCTGTGTCTCTGTGGTTTACTTTTCGTGTTTGGCGTTCTCCTCCTCCTCTTCCTCCTTCAGTTGATGAATTTGAGGATGATGATGATGATGATGATGAGGCGACCAATAGTAATATTCCAATCAGGGCAGCAAGCAAGCTCGCCATCAAGTCCTTAACCAAGAAAATATACAACAAATGTGACTCTCTTGCCATTGACAGTTGCACTATTTGTTTGGAAGAGTTTAAGAGTGGAGTCAATGTTGTCGAGTTACCCTGTGGACATGAATTTGATGATGCATGTATCGGACACTGGTTCGAGACCAATCACATTTGTCCATTGTGTCGTTTCGAGTTGCCTCGTGAGCATCATTGA

>TUE *BrLINE1-RUP*

CATCACCATCATCAAGTCATGATTATTCCTCTGTCAAGGACTTTCCATCTTCAAAACGAATGAAGTCAGAATCTGACATTTTTTCTCCTTTTATTTGAAATACATAGAGCCAAGGCCTTAGCTAGCATTGTCTGTTAATATTCCACAACTAAGTTTTTTTGTTCATGCCTTTTTGTTTTGTGGTCAATACAAAGACTGTGTTTCTGGTTTTATTATCTTGTAAGGACTCAACTACTTCCTCCAGATTCTGGCGAGGTTTTGGTGTACTACACATTGACTGTGTATGCCATTTCTCTCATGTAGATGATCAGATACCTGAGAACTTTGTGATCTTGATCAGATACACATTGACTGTGTATGGCATTTTATACTATCTTAATACATCTCTCTCATGTAGTTGATCAGATACCTGAGAACTTAGTCTTGCTCTCCCAAAGAAAGTTACATGGAGATTCCTTCTAAGGCATAGTCTCGGAAGCTGCTTCGTTATGCTATTCAAGGTTCCACTTTCGGAATAACCTTCATCATCTCACAAACATACCACTGAGCTGAAGGGAGAATGATAAGATGGAGAGAAGAGAAAACTAGAGGAAGGAGCATTATAA*TATGGTTAACTAATGTTTTAAAGAAAAAAGTCTAAAAAAAAAATCAAAAAGCTCTCCAAAAAACGTTCTCTGATCGAAACTTATTTAAATCTCCGTCACCGACTCCGGTGGTTCGCCGCTACCGGAGTCGCCCTTCTCCTTTCCTATTTTTCTTTTTTGCTTCTCTTCCATCTCCTAGCTACCTTCCGATATGCTTGTTCTTGTGGGGTTCGCTCTTCCAAGCCCTAGATCCGGCCAGATCTGAGGTACGGCAGTTGCAGGTTCCTGGAGGCACGGCGAGGAGGTGAGGCAGGTGAAGGAGGTTGAGTCGGCTCTTAGGGTGGGGAAAGATTGGTTCGGCGGTTTTGTAGATCTAGATCTGCTTGTTCGGTCGTCTTTTCCAGAAGTGGCAGCACGTGAGCTAAGGAGCTTCCTCTTCTCGGATCTGTTCCTCGGGTTTGTAGTCGGAGTGGGGTGGTGGTTCGTTTGCGGTATCCTCGCCTTGGCGTCTTGGCTATGGAGCTTCTCTTGACTCCACCCAAGGTGGGTGTCGGGGTCTATTCTCCGGTGGCGCGTCTCAGTGGCTACCTTGGTTTCGTGTGTTGGCTGGTGTGTCTCTGTCGACGGCGCGTGGGGGGTTCTCTGGCTGGTAAAGGCACGTGGAGTCGTCTATGGCTCTAGCGTCGGCTAGTCGAAGACCTTCCTCTGCCTTTGGATTTCTTCAGTTCTCGGTTTCTTCGTGTAGGAAGACCTGATTGAGATTTTCGAGGCTTCTCTTCAGGTTTACTTTGGGTCTACTGGCCTCGGTTCCGTGCCTCTGACCTGGTACTTTGCAGGCACGGTCTCGGCGGTTGGAAGATGATTCGTTTCTCTGGTTCTCAGAGGCGTAACGTGCGGTTGAGTTTGGGTTTCTACGGTCATGAGGAGACCTTCACTTCACCGGTGGCGGCGTTGTTGAGTGTTGCCATCCCAGTGGCGATTTTGATCGTCAGGTTACTCTCTTTCTCTTCCATTGTAGGTCCCGGAGTTTTCTTATCAGTGGTGCGGCTGTGACTTCAACCCGCTTTTGTATAGCTTTTTGAGAGTAGTCGCTTCCTTGCTGACTTTGTGTCGGTCTGTGGAGATGTTGTGTAAGCCTGGTGGTCTCGTTACTATTGTTGCGGCATCTGCGGGTTTCTTGGTTGTGGTTTGCTGTTTAGTTTGTTGAGCTGTAGGAGGATGTAGTAAGGCTTCGGTGTGTGGAGATGCGGCTTTCCTTTATTTGTGGGCTCCTGCTATTAAGCTTCCTTGTTTCTGGTTCTGAGAGGAGATGCGACTCCTCTTTGTTTGTGAGTTCCTGCTATTAAGCTCGGGTGATCGTTTCTGGTTCCGAGTGGAGATGCGACTCCTTCTTCTTGTGAGTTCCTGCTATTAAGCTCAGGTGATCGTTATTCTGAAGTGGAGATGCGGCTCTTCTTTGCGAGTGAGCTCCTGCTATTAAGCCCCGTTAGCTTGTGTTGTCTTGGCGTTAGTCCCATCTTGGTCCTTCGTTTTTGCTGCCATTTGAATCAGCTTCCTATGTGTTCGCAATCGGTTAGCACCCTCCTAGTGTTCTTGTATGTGTTAGCCTTGGTCGAACCTCTTCTCCGGGAATACAGTCTTTATCGCTAATTCTCTAGGTGGATGCTTACAAGTTGAAGTTTGGAGAATTACTGATTATCGTTTGTTTCCATGTTTTTATCTTTTTTTGCCGTGGAATCAATTATCCTAGGTTCTACAATTTGTTAACAACAGTCTGTAAAACCTCTTGTTCATTTTTCGAAATGATATTTACATTTTTAGCAAAAAAAAATATGGTTAACTA*TCAGCAAATATAAATGCGAGAATATCTTGAGTAATCCATATTGATTATTATATGCTTAATTACGGACAAAAATATATGATATAAATGCGGATTTTTTAAAGTAATCCACATTAACTGAATTATAAGAGAGATGCAATATTTTAATTTTGGAGTAAATTATAAGAGCGTTGTAAAATTGTGAAATTTCATGCAAGAAGATTAACTAAAACAAACGAAATCGGTCGAAAACAAAACAGTTCTTATTTGTTTTCACTTTTATATTGAACCATAATATCAATTGTATATAATATAGCGAGAATATATTTTGGAGGATTAAAACAAAAGATTGGGAGGATATAAGATTTATGAGAATATATCGCATATGACAGAATTTGAAGAATTTTTAGATGAATAACTAAATTACAATGATACAAAAAGTAATATAACATACAAAGTAAGAGCATTTTCAAAATAGCAGTCATGTTTTAATAGTGTCGATGATCATAAAAGGAAAATTGACTTTCTAAAAGGTTTGAGAAGTTATGAGAAAAGCAAAGTAGTTGGAAAAAAAACTTATATAGTCAAAATTAAACGGAAATTATATCAACAAAAAGGAAAGTAACAACAAAACCTAAATAAATTAGGTTTCAAAGATCCTCCTAGACTAAGAAATTGGTATTGTACCCCTATAAATACCCCGCCCGTATTAAACTCTACAATTCATCACTGCAATCAAACAGAACAAAGCAAAAACCCTAAGAAACTATAGATATCGTTTTTCATTATGAGATCCAGAGGCGACTTGTATTTCAGGCATGAGATCGACCGTAACCCTTCAGCCTCGGATGCTGGCACATTCAAAGCATGCGCAACCATGGTCACATGGCACGAGGACATGGAAGATTCTGAAACAACATTCTCGTTTACGTTGTCGGCGGAGGACGTCATCGAACGGCAAGGACTTGCCAACAAAATACAAGAACTAGATGAGCTATTCATGGAAGCTGCTTTCCCTCAAGAAGATAATTTGCTTTTGTTGACCCAAGAAGCCTATCATTGTTTCATTGAAGTGATTTCCTCAAGTGATTATAGCAGAGATTGTGCTGTGTCTCTGTGGTTTACTTTTCGTGTTTGGCGTTCTCCTCCTCCTCTTCCTCCTTCAGTTGATGAATTTGAGGATGATGATGATGATGATGATGAGGCGACCAATAGTAATATTCCAATCAGGGCAGCAAGCAAGCTCGCCATCAAGTCCTTAACCAAGAAAATATACAACAAATGTGACTCTCTTGCCATTGACAGTTGCACTATTTGTTTGGAAGAGTTTAAGAGTGGAGTCAATGTTGTCGAGTTACCCTGTGGACATGAATTTGATGATGCATGTATCGGACACTGGTTCGAGACCAATCACATTTGTCCATTGTGTCGTTTCGAGTTGCCTCGTGAGCATCATTGA

>WTC *BrRUP*

CCTCATCATCATCAAGTCATGATGATTCCTCTGTCAAGGACTTTCCATCTTCAAAACGAATGAAGTCAGAATCTGACATTTTTTCTCCTTTCATTTGAAATACTTAGAGCCAAGACCTTAGCTAGCATTGCCTGTTAATATTCCACAACTAAGTTTTTCTGTTCATGCCTTTTTGTTTTGTGGTCAATACAAAGACTGTGTTTCTGGTTTTGTTATCTTGTAAGAACTCAACTACTTCCTCCAGATTCTGGCGAGGGTTTGGTGTACTACACATTGACTGTGTATGCCATTTCCTCATGTAGTTGATCAGATACCTGAGAACTTAGTGATCTTGATCAGATACACATTGACTGTGTATGGCATTTTATACTATCTTAATACATCTCTCTCATGTAGTTGATCAGATACCTGAGAACTTAGTCTTGCTCTCCCAAAGAAAGTTACATGGAGATTCCTTTTAAGGCATAGTCTCGGAAGCTGCTTCGTTATGCTATTCAAGGTTCCACTTTCGGAATAACCTTCATCATCACAAACATACCACTGAGCTGAAGGGAGAATGATAAGATGGAGAGAAGAGAAAACTAGAGGAAGGACTAAGGAGCATTATATGGTTAACTATCAGCAAAAGTATACAAATATAAATGCGAGAATATCTTGAGTAATCCATATTGATTATATTATATGTTTAATTATGGACAAAAATATTTGATATAAATGCGGATTTTTGAAGTAATCCACATTAACTGAATTATAAGAGCGATGCAATATTTTAATTTTGGACTAAATTATAAGAGCGTTGTAAAATTGTGAAATTTCATGCAAGAAGATTAACTAAAACAAACGAAATCGGTGGAAAACAAAACAGTTCTTATTTGTTTTCACTTTTATATTGAACCATAATATCAATTGTATACTATAATATAGCGAGAATATATTTTGGAGGAATTAAAACAAAAGATTGGGAGGATATAAGATTTATGAGAATATCTCGCATATGACAGAATTTGAAGAATTTTTAGATGAATAACTAAATTACAATGATACAAAAAGTAATATAACATACAAAGCAAGAGCATTTTCAAAATAGCAATCATGTTTTAATAGTGTCGATGATCATAAAAGGAAAATTGACTTTCTAAAAGGTTTGAGAAGTTATTAGAAAAGCAAAGTAGTTGAAAAAAAAACTTATATAGTCAAAATTAAACGGAAATTATATCAACAAAAGGGAAAGTAACAACAAAACCTAAATAAATTAGTATGATCTTGTTTCTAAAAAAACCCTAAGAAACTATAGATATCGTTTTTCATTATGAGATCTAGAGGCGACTTGTATTTCAGGCATGAGATCGACCGTAACCCTTAAGCCTCGGATGCTGGCACAATCAAAGCATGCGCAACCATGGTCACATGGCAGGAGGACATGGCAGATTTTGAAACAACATTCTCGTTTACGTTGTCGGCGGAGGACGTCATCGAACGCCAAAGACTTGCCAACAAAATACAAGAACTAGATGAGTTATTCATGGAATCTGCTTTCCCTCGAGAAGATAATTTGCTTTTGTTGACCCAAGAAGCCTATCATTGTTTCATTAAATTGATTTCCTCACGTGATTATAGTAGAGATTGTGTTATGTCTCTGTGGTTTACTTTTCGTGTTTGGCGTACTCCTCTTCCTCCTTCAATTGATGAATGTGAGGATGATGATGATGATGATGAGGCGACCAATAGTAATATTCCAATCAGGACAGCAAGCAAGCTCGCGGTCAAGTCCTTAACCAAGAAAATATACAACAAAGGTGACTCTCTCGCCATTGACAGTTGCACTATTTATACCCGGTTGGAAGGGTTAATCACGACCTTTATATCCGGTCGGAAGGGTTAA

Figure S2 Comparative sequence analysis of *BrLINE1-RUP* and *BrRUP* in 18 representative *B. rapa* genomes

Note:

The upstream region from start codon (ATG) of *BrRUP*;

Coding sequences of *BrRUP*;

*Transposable element*;

*Open reading frame of transposable element*;

*130-bp fragment of transposable element* *identical with the 130-bp insertion fragment in Brcer2 of HN19-G*;

*Poly(A)*;

*Target site duplication in BrLINE1-RUP*
