# Supplementary material for: A BrLINE1-RUP insertion in BrCER2 alters cuticular wax biosynthesis in Chinese cabbage (Brassica rapa L. ssp. pekinensis)
Source: Front Plant Sci. 2023 Jul 12;14:1212528. doi: 10.3389/fpls.2023.1212528 (PMC10368883; doi:10.3389/fpls.2023.1212528)
Supplement: Supplementary file 5 [file DataSheet_5.doc]

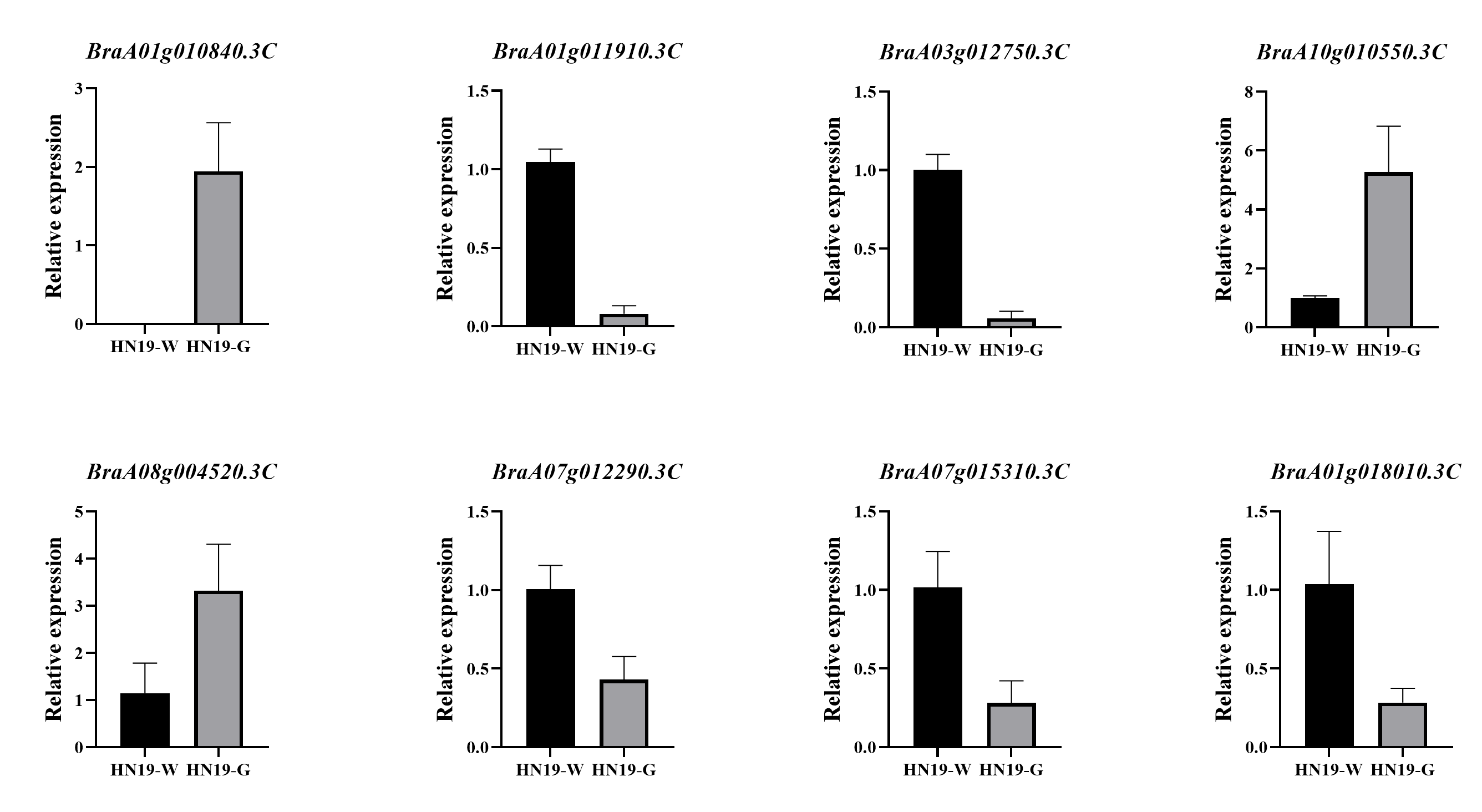


FIGURE S4 Expression validation of RNA-Seq data by qRT-PCR. Error bars indicate SD (*n*=3). Y-axis represents expression level relative to HN19-W.
